# Semaglutide on liver fibrosis and heart outcomes in patients at high risk of liver fibrosis: a prespecified analysis of the SELECT randomized trial

**DOI:** 10.1038/s41591-026-04281-1

**Published:** 2026-04-02

**Authors:** Sebastian M. Meyhöfer, Bertrand Cariou, Cintia Cercato, Helen M. Colhoun, John Deanfield, Michelle T. Long, Ole Kleist Jeppesen, A. Michael Lincoff, Ildiko Lingvay, Jorge Plutzky, Philip N. Newsome, Stephen J. Nicholls, Maria Quiroga, Ferruccio Santini, Arun J. Sanyal, Steven E. Kahn

**Affiliations:** 1 Clinical, Medical & Regulatory, Novo Nordisk Pharma GmbH, Mainz, Germany; 2 Department of Internal Medicine 1 – Endocrinology & Diabetes, University Medical Centre Lübeck, Lübeck, Germany; 3 Nantes Université, CHU Nantes, CNRS, INSERM, l’institut du thorax, Nantes, France; 4 Obesity Unit, Department of Endocrinology, Clinical Hospital of the University of São Paulo, São Paulo, Brazil; 5 Institute of Genetics and Cancer, The University of Edinburgh, Edinburgh, UK; 6 Institute of Cardiovascular Science, University College London, London, UK; 7 Novo Nordisk A/S, Søborg, Denmark; 8 Department of Medicine, Section of Gastroenterology, Boston University Chobanian and Avedisian School of Medicine, Boston, MA USA; 9 Department of Cardiovascular Medicine, Cleveland Clinic and Cleveland Clinic Lerner College of Medicine of Case Western Reserve University, Cleveland, OH USA; 10 Department of Internal Medicine/Endocrinology and Peter O’Donnell Jr. School of Public Health, University of Texas Southwestern Medical Center, Dallas, TX USA; 11 Cardiovascular Division, Brigham and Women’s Hospital, Harvard Medical School, Boston, MA USA; 12 Roger Williams Institute of Liver Studies, School of Immunology and Microbial Sciences, Faculty of Life Sciences and Medicine, King’s College London, Foundation for Liver Research and King’s College Hospital, London, UK; 13 Victorian Heart Institute, Monash University, Melbourne, Victoria Australia; 14 Obesity and Lipodystrophy Centre, University Hospital of Pisa, Pisa, Italy; 15 Division of Gastroenterology, Hepatology and Nutrition, Virginia Commonwealth University School of Medicine, Richmond, VA USA; 16 Department of Medicine, VA Puget Sound Health Care System and University of Washington, Seattle, WA USA; 17 Pennington Biomedical Research Center, Baton Rouge, LA USA; 18 Department of Biostatistics, University of Washington, Seattle, WA USA; 19 Northwestern University Feinberg School of Medicine, Northwestern University, Chicago, IL USA

**Keywords:** Cardiovascular diseases, Medical research

## Abstract

In the SELECT trial, once-weekly subcutaneous semaglutide reduced major adverse cardiovascular events (MACE) by 20% versus placebo in patients with atherosclerotic cardiovascular disease and obesity but without diabetes. We examined semaglutide in SELECT patients at high risk for substantial liver fibrosis in a prespecified secondary analysis. Liver biochemical tests and steatosis risk according to fatty liver index were assessed over 104 weeks. Subgroup analyses of the primary MACE (a composite endpoint including cardiovascular death, nonfatal myocardial infarction or nonfatal stroke) outcome used baseline Fibrosis-4 scores ≥ 1.3, age-specific (≥1.3 (<65 years) or ≥2.0 (≥65 years)) and any age with Fibrosis-4 > 2.67. MACE was reduced by 26% (hazard ratio (HR) 0.74; 95% confidence interval (CI) 0.63–0.88; *P* = 0.0004), 21% (HR 0.79; 95% CI 0.63–0.98; *P* = 0.035) and 34% (HR 0.66; 95% CI 0.39–1.10; *P* = 0.11), respectively. Semaglutide led to a 28% greater decrease in fatty liver index versus placebo (HR 0.72; 95% CI 0.71–0.73; *P* < 0.0001). In conclusion, semaglutide reduced MACE versus placebo in patients at risk for substantial liver fibrosis, as seen in the overall SELECT population. ClinicalTrials.gov registration no. NCT03574597

## Abstract (web summary)

A prespecified analysis from the SELECT trial showed that semaglutide reduces major adverse cardiovascular events by 20% compared with placebo, particularly in patients at high risk of fibrosis, as indicated by the Fibrosis-4 index.

## Main

The coexistence of overweight and obesity with metabolic dysfunction-associated steatotic liver disease (MASLD) underscores a substantial and intricate interplay between metabolic dysregulation and hepatic pathology. Overweight and obesity are established risk factors for the development and progression of MASLD, contributing to the accumulation of hepatic triglycerides and the subsequent transition from simple steatosis to metabolic dysfunction-associated steatohepatitis (MASH) and advanced fibrosis^1,2^. This overlap reflects the bidirectional relationship between metabolic syndrome and hepatic steatosis, with adipose tissue dysfunction, systemic insulin resistance and subclinical inflammation having pivotal roles in the pathogenesis of MASLD and progression to fibrotic MASH and cirrhosis^3^. The global prevalence of MASLD is estimated to be ~30% and has risen in parallel with the global surge in obesity, making it an increasingly recognized health concern^4,5^.

MASH contributes as an independent risk factor for the development and progression of atherosclerosis and atherosclerotic cardiovascular disease (ASCVD)^1,6^. The severity of hepatic steatosis and fibrosis in MASLD/MASH appears to significantly impact the cardiovascular (CV) risk profile, with a direct association between the degree of steatosis or fibrosis and the incidence of major adverse CV events (MACE)^7,8^. The interplay between MASLD/MASH, hepatic steatosis and fibrosis, and MACE underscores the need for a comprehensive risk assessment tailored to this high-risk population and is pivotal to refining therapeutic interventions aimed at mitigating the dual burden of hepatic and CV morbidity and mortality.

The diagnosis of MASH has historically relied on liver biopsy, allowing for the direct histological assessment of key MASH aspects such as steatosis, inflammation and staging of fibrosis. However, the invasiveness and potential sampling variability of liver biopsy have spurred the search for noninvasive diagnostic modalities^9,10^. The Fibrosis-4 (FIB-4) index, calculated from readily available measures (age, aspartate aminotransferase (AST), alanine aminotransferase (ALT) and platelet count), has emerged as a validated tool for identifying individuals at risk of fibrosis^10^. This offers a pragmatic alternative to liver biopsy in a clinical setting for patients with metabolic risk factors. The FIB-4 index enables risk stratification of low (<1.3), intermediate (1.3–2.67) and high risk (>2.67) for advanced fibrosis and major adverse liver events^1^ and further correlates with MACE^8^. As age is a factor in the FIB-4 equation, an age-adjusted higher cutoff of 2.0 in individuals older than 65 years was applied to avoid overestimation of advanced liver fibrosis risk^11^. These cutoff values allow for increased accuracy in advanced MASH fibrosis risk stratification.

In the SELECT CV outcome trial, which included patients aged 45 years or older with a body mass index (BMI) ≥ 27.0 kg m^−^^2^ and established ASCVD but without diabetes, those treated with the glucagon-like peptide-1 receptor agonist (GLP-1RA), semaglutide 2.4 mg, had a 20% MACE reduction compared with those receiving placebo on top of standard of care^12^. The aim of this prespecified analysis was to evaluate the impact of semaglutide on liver enzymes and suspected MASLD in patients from the SELECT trial, and to explore the CV benefits of semaglutide in a subgroup at risk for substantial fibrosis as defined by FIB-4.

## Results

FIB-4 score data were available for 16,585 patients (94.2% of the total SELECT population). All patients were stratified according to FIB-4 score for their predicted risk of substantial liver fibrosis: 6,567 patients (37.3% of all) with an FIB-4 score ≥ 1.3, 3,664 patients (20.8% of all) either aged <65 years with an FIB-4 score ≥ 1.3 or aged ≥65 years with an FIB-4 score ≥ 2.0 and 509 (2.9% of all) patients with predicted substantial fibrosis (an FIB-4 score > 2.67 irrespective of age) were included in this analysis. As summarized in Table 1, the baseline characteristics of the respective subgroups were generally similar between the semaglutide and placebo groups, respectively. Notably, the use of statins was slightly lower in the FIB-4 > 2.67 subgroup (85.5%) compared with the other FIB-4 subgroups (88.9% for FIB-4 ≥ 1.3 and 89.3% for the age-dependent FIB-4 subgroup), but similar between semaglutide and placebo groups.

**Table 1:** Baseline characteristics according to FIB-4 stratification

Characteristics	FIB-4 ≥ 1.3			FIB-4 ≥ 1.3/≥ 2.0 according to age (<65 years/≥65 years)		FIB-4 > 2.67 (any age)	
	Semaglutide (*n* = 3,293)	Placebo (*n* = 3,274)	Semaglutide (*n* = 1,833)		Placebo (*n* = 1,831)	Semaglutide (*n* = 246)	Placebo (*n* = 263)
Age, years	66.7 (7.9)	66.6 (7.8)	63.5 (8.4)		63.4 (8.2)	71.8 (8.1)	70.5 (8.6)
Female, *n* (%)	765 (23.2)	743 (22.7)	354 (19.3)		350 (19.1)	37 (15.0)	59 (22.7)
Body weight, kg	95.2 (16.9)	95.5 (17.3)	96.7 (17.6)		96.8 (17.6)	94.2 (17.8)	94.1 (18.8)
BMI, kg m^−^^2^	32.8 (4.7)	32.8 (4.7)	32.9 (4.9)		33.0 (4.8)	32.6 (5.3)	32.4 (4.7)
Waist circumference, cm	111.0 (12.5)	111.1 (12.6)	111.2 (12.7)		111.0 (12.6)	111.6 (12.6)	110.9 (12.9)
Systolic blood pressure, mmHg	131.5 (16.0)	131.7 (15.5)	131.3 (16.1)		130.9 (15.4)	131.4 (16.7)	132.4 (16.1)
Diastolic blood pressure, mmHg	77.8 (10.1)	77.9 (10.0)	78.6 (10.1)		78.6 (9.8)	76.1 (10.4)	76.2 (10.2)
HbA_1c_, %	5.8 (0.3)	5.8 (0.3)	5.7 (0.3)		5.7 (0.3)	5.7 (0.4)	5.7 (0.4)
Pre-diabetes, *n* (%)	2,061 (62.6)	2,033 (62.1)	1,120 (61.1)		1,108 (60.5)	147 (59.8)	145 (55.1)
Triglycerides, mg dl^−^^1^, median (IQR)	125 (93–174)	127 (94–181)	125 (93–176)		129 (93–184)	115 (87–164)	118 (85–174)
CVD history							
≥2 CVDs, *n* (%)^a^	345 (10.5)	346 (10.6)	175 (9.5)		159 (8.7)	40 (16.3)	33 (12.5)
Only MI	2,217 (67.3)	2,203 (67.3)	1,271 (69.3)		1,293 (70.6)	159 (64.6)	173 (65.8)
Only stroke	531 (16.1)	516 (15.8)	284 (15.5)		266 (14.5)	34 (13.8)	38 (14.4)
Only PAD	129 (3.9)	128 (3.9)	63 (3.4)		64 (3.5)	7 (2.8)	10 (3.8)
MI + stroke	165 (5.0)	163 (5.0)	82 (4.5)		84 (4.6)	20 (8.1)	18 (6.8)
MI + PAD	128 (4.0)	121 (3.7)	71 (3.9)		56 (3.1)	16 (6.5)	8 (3.0)
Stroke + PAD	28 (0.9)	37 (1.1)	11 (0.6)		11 (0.6)	2 (0.8)	6 (2.3)
MI + stroke + PAD	17 (0.5)	20 (0.6)	7 (0.4)		6 (0.3)	0	1 (0.4)
Other	77 (2.3)	86 (2.6)	44 (2.4)		51 (2.8)	8 (3.3)	9 (3.4)
FIB-4 score distribution							
Mean (s.d.)	1.84 (0.63)	1.86 (0.80)	2.01 (0.73)		2.04 (0.98)	3.46 (1.07)	3.65 (1.80)
Median (IQR)	1.66 (1.46–2.01)	1.65 (1.45–2.01)	1.84 (1.47–2.30)		1.80 (1.47–2.31)	3.12 (2.86–3.67)	3.16 (2.84–3.77)
Geometric mean ALT level (CoV), U l^−1^	23.3 (60.4)	23.2 (59.8)	25.1 (67.3)		25.1 (65.5)	23.2 (92.0)	23.9 (93.3)
Geometric mean AST level (CoV), U l^−1^	25.1 (37.7)	25.1 (38.6)	27.6 (40.8)		27.6 (41.5)	32.8 (56.8)	34.5 (61.3)
Geometric mean GGT (CoV), U l^−1^	42.2 (61.9)	43.5 (67.6)	49.2 (77.9)		50.1 (84.8)	72.0 (144.5)	82.8 (172.6)
Thrombocytes, 10^9^ per liter	194.8 (22.4)	194.5 (23.0)	181.5 (23.2)		180.6 (23.4)	146.4 (32.3)	143.3 (32.6)
Medications, *n* (%)							
Statins	2,914 (88.5)	2,925 (89.3)	1,625 (88.7)		1,646 (89.9)	211 (85.8)	224 (85.2)

Data are shown as the mean (±s.d.) unless otherwise stated.

^a^Patient numbers for MI + stroke, MI + PAD, stroke + PAD and MI + stroke + PAD do not add up to the patient number presented for ≥2 CVDs because of differences in how missing data were handled for the individual components and ≥2 CVDs. CoV, coefficient of variation in %; HbA_1c_, glycated hemoglobin; IQR, interquartile range; MI, myocardial infarction; PAD, peripheral artery disease.

### Cardiovascular outcomes

**Fig. 1:**
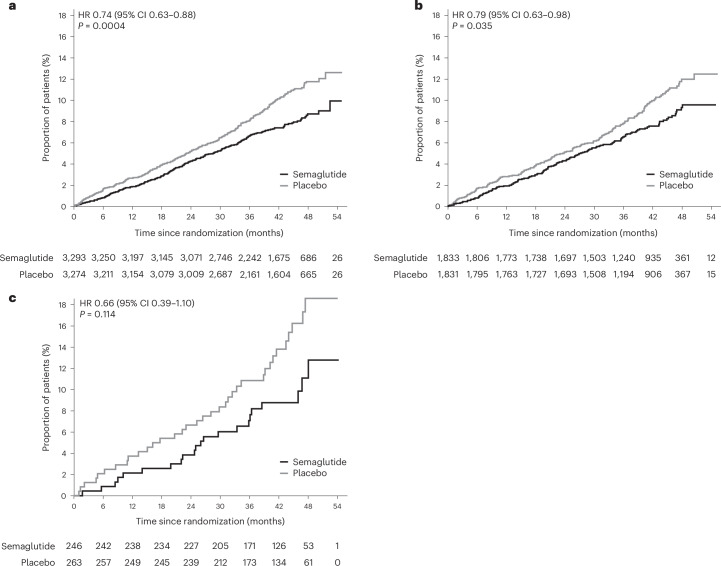
Cumulative incidence of MACE in patients at risk for significant fibrosis according to respective FIB-4 stratification. Data from the in-trial period. **a**, FIB-4 ≥ 1.3 (intermediate risk). **b**, FIB-4 ≥ 1.3/≥ 2.0 (intermediate risk according to age <65 years/≥65 years). **c**, FIB-4 > 2.67 (high risk irrespective of age). Cumulative incidence estimates are based on time from randomization to first EAC-confirmed MACE with non-CV death modeled as competing risk using the Aalen–Johansen estimator. *P* value: two-sided *P* value for test of no difference. Patients without events of interest were censored at the end of their in-trial observation period. Values underneath the figures are the number of patients. EAC, event adjudication committee. Source data

In the cohort with an FIB-4 score ≥ 1.3, the primary MACE endpoint occurred in 244 of 3,233 patients (7.5%) in the semaglutide group and in 321 of 3,274 patients (9.8%) in the placebo group (HR 0.74; 95% CI 0.63–0.88; *P* = 0.0004; Fig. 1a).

In the second subgroup with an FIB-4 score ≥ 1.3 or aged ≥65 years with an FIB-4 score ≥ 2.0, the primary MACE endpoint occurred in 140 of 1,833 patients (7.6%) in the semaglutide group and in 176 of 1,831 patients (9.6%) in the placebo group (HR 0.79; 95% CI 0.63–0.98; *P* = 0.035; Fig. 1b).

**Fig. 2:**
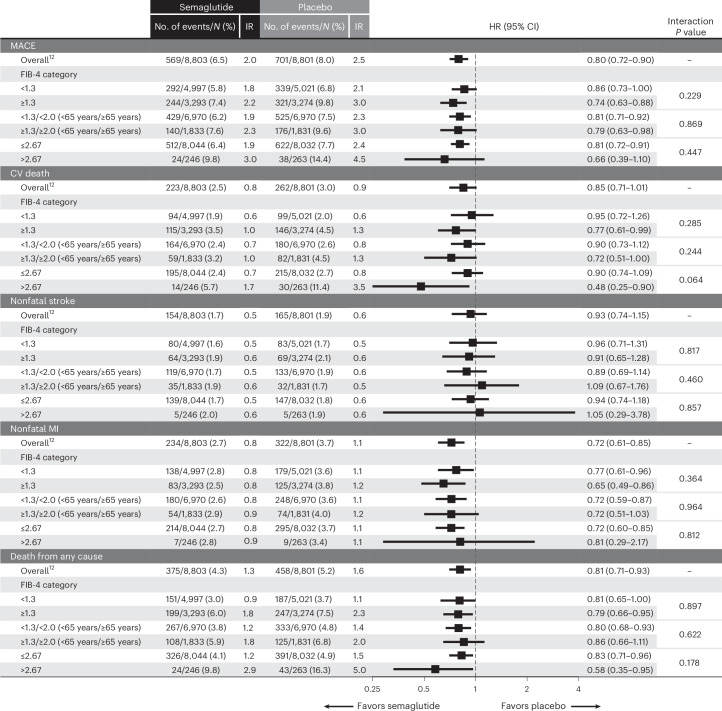
HRs for the components of MACE and on death from any cause. Values shown in the forest plot are HRs and 95% CIs. *P* value for test of no interaction effect. IR, incidence rate; *N*, analyzed patients. Source data

In the third subgroup of 475 patients with suspected substantial fibrosis (FIB-4 score > 2.67 irrespective of age), the primary MACE endpoint occurred in 24 of 246 patients (9.8%) treated with semaglutide compared with 38 of 263 patients treated with placebo (14.4%), yielding an HR of 0.66 (95% CI 0.39–1.10; *P* = 0.11; Fig. 1c). The HRs and incidence rates of each respective component of MACE taken individually and on death from any cause are depicted in Fig. 2.

**Extended Data Fig. 1:**
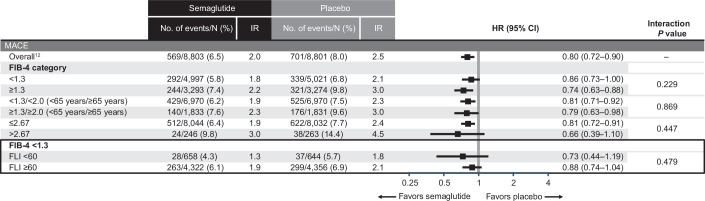
Time from randomization to first MACE by change in FLI. Values shown in forest plot are HR and 95% CIs. *P* value for test of no interaction effect. CI, confidence interval; CV, cardiovascular; FIB-4, Fibrosis-4; FLI, fatty liver index; HR, hazard ratio; IR, incidence rate; MACE, major adverse cardiovascular events; MI, myocardial infarction; N, analyzed patients. Source data

The primary MACE endpoint was also assessed using the fatty liver index (FLI) in patients with or without suspected MASLD (FLI ≥ 60 or <60, respectively) and without fibrosis (FIB-4 < 1.3), with no interaction effect observed between treatment groups in those with an FLI < 60 (HR 0.73; 95% CI 0.44–1.19) compared with those with an FLI ≥ 60 (HR 0.88; 95% CI 0.74–1.04; *P* = 0.48; Extended Data Fig. 1). Additionally, adjusting for changes in body weight and glycated hemoglobin in a time-dependent Cox regression suggested that these variables had a negligible influence on the effect of semaglutide on the primary MACE outcome (Extended Data Tables 1 and 2).

### Liver measures and indices

**Fig. 3:**
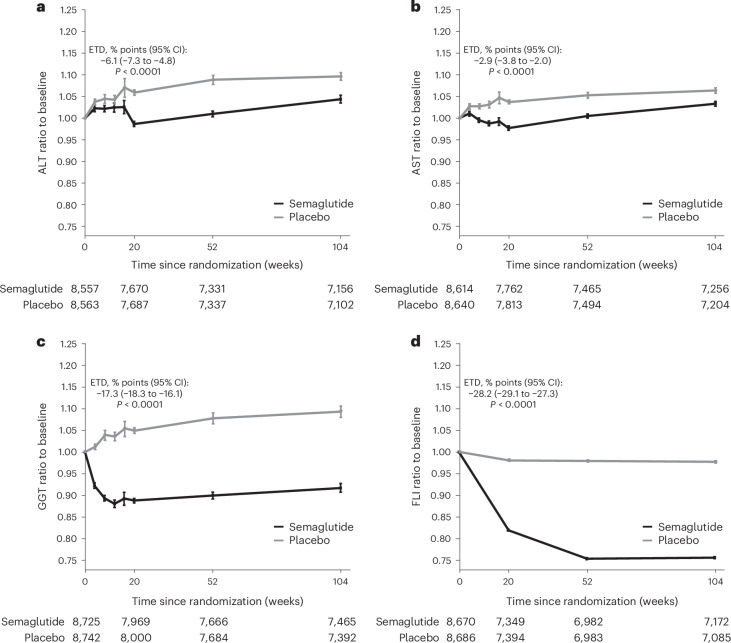
Changes in liver parameters over time according to treatment group. **a**–**d**, Ratio to baseline and standard errors of ALT (**a**), AST (**b**), GGT (**c**) and FLI (**d**) at baseline, and weeks 26, 52 and 104. Observed data from the in-trial period. Error bars are ±s.e. of the mean on the logarithmic scale back-transformed to natural scale with the exponential. *P* value: two-sided *P* value for test of no difference. Exact *P* values: *P* = 6.180993 × 10^−21^ (**a**); *P* = 3.497464 × 10^−10^ (**b**); *P* = 1.01892 × 10^−170^ (**c**); *P* < 0.0000000000000001 (**d**). The numbers shown below the graphs represent the number of patients contributing to the means. Measurements at weeks 4–16 are for a subpopulation of 6,011 patients from European countries in which extra laboratory measurements were mandated by safety guidance (Austria, Belgium, Czech Republic, Denmark, Finland, France, Germany, Greece, Hungary, Republic of Ireland, Italy, Latvia, the Netherlands, Norway, Poland, Portugal, Romania, Spain, Sweden and the UK). ETD, estimated treatment difference. Source data

Baseline characteristics for liver parameters and indices from the whole SELECT population are depicted in Table 2. From baseline values within the normal range, ALT, AST and gamma-glutamyl transferase (GGT) levels declined after initiation of semaglutide treatment, reaching a nadir at approximately 20 weeks, after which ALT and AST levels increased back toward the pretreatment baseline over time while GGT levels remained relatively stable (Fig. 3a–c). Mean ± standard deviation (s.d.) changes from baseline of ALT, AST and GGT levels upon treatment with semaglutide versus placebo were −1.83 ± 0.24 versus 0.07 ± 0.26 U l^−1^, −0.45 ± 0.15 versus 0.47 ± 0.16 U l^−1^ and −7.18 ± 0.47 versus 0.99 ± 0.50 U l^−1^, respectively. The corresponding estimated treatment difference versus placebo at week 104 was −1.9 U l^−1^ (95% CI −2.6 to −1.2; *P* < 0.0001), −0.9 U l^−1^ (95% CI −1.4 to −0.5; *P* < 0.0001) and −8.2 U l^−1^ (95% CI −9.5 to −6.8; *P* < 0.0001), respectively. The relative decrease from baseline at week 104 with semaglutide versus placebo treatment was 6% (95% CI 0.93–0.95; *P* < 0.0001) for ALT, 3% (95% CI 0.96–0.98; *P* < 0.0001) for AST and 17% (95% CI 0.82–0.84; *P* < 0.0001) for GGT. Mean ± s.d. FLI decreased by 18.63 ± 0.21 with semaglutide treatment versus 2.49 ± 0.19 with placebo (Fig. 3d), representing a treatment difference of 16.1 (95% CI −16.7 to −15.6; *P* < 0.0001), thereby showing a 28% greater decrease in FLI (95% CI 0.71–0.73; *P* < 0.0001) with semaglutide versus placebo. A higher proportion of patients treated with semaglutide showed improvement from baseline FLI compared with placebo (26.1% versus 11.6%, respectively), and a lower proportion showed worsening of FLI (3.0% versus 7.8%, respectively). As reported in the primary analysis of SELECT, semaglutide treatment also resulted in a −9.39% mean change in body weight over 104 weeks versus −0.88% with placebo (*P* < 0.0001)^12^.

**Table 2:** Baseline characteristics of biochemical liver tests

Characteristic	Semaglutide (*n* = 8,803)	Placebo (*n* = 8,801)	Overall (*N* = 17,604)
ALT, *n*^a^			
<24 U l^−1^	4,237	4,273	8,510
≥24 U l^−1^	4,320	4,290	8,610
Geometric mean (CoV), U l^−1^	24.0 (53.6)	24.0 (53.3)	24.0 (53.4)
AST, *n*^a^			
<22 U l^−1^	4,265	4,290	8,555
≥22 U l^−1^	4,349	4,350	8,699
Geometric mean (CoV), U l^−1^	22.3 (34.4)	22.3 (34.8)	22.3 (34.6)
GGT, *n*^a^			
<30 U l^−1^	4,694	4,689	9,383
30– < 60 U l^−1^	2,846	2,854	5,700
≥60 U l^−1^	1,185	1,199	2,384
Geometric mean (CoV), U l^−1^	30.8 (69.4)	30.8 (69.8)	30.8 (69.6)
FLI, *n*^a^			
<85	4,247	4,291	8,538
≥85	4,423	4,395	8,818
Geometric mean (CoV)	77.7 (27.6)	77.9 (27.2)	77.8 (27.4)
FIB-4 index, *n*^a^			
≥1.3	3,293	3,274	6,567
≥1.3/≥ 2.0 according to age (< 65/≥ 65 years)	1,833	1,831	3,664
>2.67	246	263	509
Geometric mean (CoV)	1.2 (44.2)	1.2 (44.1)	1.2 (44.1)

Full analysis set. ^a^Data available for 17,120 (ALT), 17,254 (AST), 17,467 (GGT), 17,356 (FLI) and 16,585 (FIB-4) patients at baseline.

## Discussion

This prespecified analysis of the SELECT trial yielded insights into the hepatic benefits of semaglutide treatment in individuals with overweight or obesity and established ASCVD but without diabetes, and established the cardioprotective benefits of semaglutide in the subgroup with high likelihood of substantial liver fibrosis based on the FIB-4 index. Within the SELECT population, there is a clinically notable proportion of approximately one of five patients at concomitant risk for substantial fibrosis as predicted by the FIB-4. As shown here, the primary results from the SELECT trial of a 20% MACE reduction in patients with established ASCVD and overweight or obesity^12^ is also seen in the subgroup of these patients with concurrent MASLD and at risk for substantial fibrosis. Further corroborating its metabolic benefits, parameters of liver health and steatosis improved significantly with semaglutide treatment in this population. Our comprehensive analysis, although hypothesis-generating, provides valuable clinical implications, offering a platform for understanding the potential of semaglutide as a multifaceted therapeutic agent.

Semaglutide 2.4 mg is currently under investigation for the specific treatment of MASH in patients with MASH and F2–F3 fibrosis in the phase III ESSENCE trial. Recently, results from the first 72-week interim analysis of 800 patients in ESSENCE showed that semaglutide 2.4 mg leads to significant resolution of MASH, improvement in liver fibrosis and improvements in cardiometabolic risk factors compared with placebo^13^. Moreover, improvements in AST and ALT levels with semaglutide versus placebo were apparent as early as week 12 (ref. ^13^). How this translates or contributes to the CV benefits of semaglutide in patients with MASH remains to be shown during the course of this ongoing phase III trial.

Recognizing the current knowledge on incretin-based therapies in metabolic disease, clinical practice guidelines for the treatment of people living with MASH already recommend optimizing the management of respective comorbidities such as type 2 diabetes and obesity by treatment with GLP-1RAs^1^. Therefore, the findings of our current analysis are relevant, shedding light on the potential of semaglutide in mitigating CV risk in individuals with overweight or obesity and established ASCVD and concurrent MASLD, at least to the same extent as in patients with overweight or obesity and ASCVD, regardless of MASH.

When interpreting these results from a mechanistic perspective, there are commonly shared pathways driving both ASCVD and MASH. Adipose tissue dysfunction, systemic insulin resistance and subclinical inflammation at the systemic and tissue levels are established pathophysiological key drivers for both ASCVD^14–16^ and MASH^17–19^. In turn, GLP-1RA treatment, directly and indirectly, addresses these pathways by reducing visceral fat mass and improving adipose tissue health and glucose homeostasis^20^. This substantially reduces the flux of free fatty acids and lipotoxic lipids, and improves glucose metabolism in the liver, along with decreasing inflammation and restoring normal adipokine levels^21^. Semaglutide has been shown to reduce markers of systemic inflammation, for example, high-sensitivity C-reactive protein^22^ and tissue-specific inflammation in the liver^23^ and heart^24–26^, and increases the secretion of adiponectin, an insulin-sensitizing adipokine^27^.

In addition to the CV outcomes, this analysis unveiled that semaglutide treatment significantly improved liver enzymes and FLI. The observed reductions in ALT, AST and GGT levels, along with the substantial decrease in FLI, underscore the potential of semaglutide not only in addressing CV risk but also its potential in concurrently ameliorating liver injury in people with overweight or obesity but without diabetes. Furthermore, the significant weight loss induced by semaglutide^12^ aligns with the known benefits of weight reduction in liver steatosis and inflammation^28,29^. Sustained weight loss ameliorates parameters of liver health in a dose-dependent manner; therefore, it is recommended by the EASL–EASD–EASO Clinical Practice Guidelines on the management of MASLD^1^. Whether achieved by dietary means, pharmacological intervention or bariatric surgery^30–33^, there is evidence that a body weight reduction of 5% or more is required to reduce liver lipid content, 7–10% to improve hepatic inflammation and 10% or more to improve fibrosis^1^. It is worth noting that in SELECT, the beneficial effects on ALT, AST and GGT levels occurred early after initiation of semaglutide, a similar pattern seen with glycemia^34^, but that maximal weight loss did not occur until beyond 52 weeks^12^, suggesting the impact on liver enzymes was mediated by more than just weight loss. The biology of weight loss after dietary intervention versus medical treatment versus bariatric surgery is different; therefore, the mode of weight loss may have implications for the clinical benefits.

Although the findings of this analysis present compelling evidence for the clinical relevance of semaglutide in addressing ASCVD and MASLD in individuals living with overweight or obesity, it is essential to acknowledge potential methodological limitations. Longitudinal studies assessing the sustained impact of semaglutide on liver health and CV outcomes are imperative (and ongoing) to elucidate its long-term benefits and inform optimal treatment durations. Although the observed MACE reduction with semaglutide treatment in this population at high risk for further CV events is acknowledged, the clinical relevance of improvement in biochemical liver tests within the normal range with GLP-1RAs is less evident^35^. While the subgroup with an FIB-4 ≥ 1.3 showed substantially lower incidence of CV events with semaglutide versus placebo, this pattern was observed as a trend across intermediate-risk and high-risk FIB-4 groups (FIB-4 < 1.3 versus ≥1.3 and FIB-4 > 2.67 versus FIB-4 ≤ 2.67). As this analysis was not powered to reach significance because of the subcategory population sizes in the intermediate-risk and high-risk FIB-4 groups, further investigations into these populations are needed. Additionally, noninvasive surrogate measures to determine liver health (such as FIB-4 and FLI) are limited in diagnostic accuracy and may need to be considered in complement to liver biopsy and histological techniques^36^.

In summary, the improvements in CV outcomes associated with semaglutide treatment highlight its potential as a therapeutic option for individuals with overweight or obesity and established ASCVD and concurrent MASH. Furthermore, hepatic parameters improving with semaglutide treatment in the whole SELECT population, irrespective of MASH, offers a rationale for the integration of comprehensive management strategies that address the interconnected burden of hepatic and CV complications in people with overweight and obesity.

## Methods

### Study design and patients

The protocol for SELECT was approved by the institutional review board and ethics committee at each participating center^12^. All patients provided written informed consent before commencement of any trial-specific activity.

The current work reports a prespecified analysis of SELECT, a randomized, double-blind, placebo-controlled, event-driven CV outcome trial (NCT03574597), details of which have been reported previously^12,37,38^. The trial evaluated once-weekly subcutaneous semaglutide 2.4 mg versus placebo in reducing the risk of MACE (a composite endpoint comprising CV death, nonfatal MI or nonfatal stroke) in individuals with established ASCVD and overweight or obesity but without diabetes.

Eligible patients were aged ≥45 years with a BMI of ≥27 kg m^−^^2^ and established ASCVD, defined as at least one of the following: prior MI, prior ischemic or hemorrhagic stroke, or symptomatic peripheral artery disease). Exclusion criteria included prior MI or stroke, hospitalization for unstable angina pectoris or a transient ischemic attack, all within 60 days of screening; glycated hemoglobin ≥6.5% (48 mmol mol^−1^); history of type 1 or 2 diabetes; New York Heart Association class IV heart failure; presence of end-stage kidney disease; or need for chronic or intermittent dialysis. A total of 17,604 patients were randomized (1:1) to escalating doses of once-weekly subcutaneous semaglutide over 16 weeks to a target dose of 2.4 mg (*n* = 8,803) or placebo (8,801)^38^. The mean (±s.d.) duration of exposure to semaglutide or placebo in the SELECT trial was 34.2 (13.7) months; the mean (±s.d.) duration of follow-up was 39.8 (9.4) months^12^.

This prespecified analysis explored the effect of semaglutide on the primary composite endpoint of MACE for patients at risk for substantial fibrosis as indicated by an FIB-4 score of 1.3 or greater. To adjust for patients’ ages, the analysis was also performed in a subgroup with an FIB-4 score of 1.3 or greater for patients aged less than 65 years and an FIB-4 score of 2.0 or greater for patients aged 65 years or older (FIB-4 age-specific) and for a further subgroup of patients of any age with an FIB-4 score greater than 2.67. After review of medical history and co-medication, patients with an etiology of liver disease other than MASH were not included in this analysis.

In addition, the aim of this analysis was to assess the impact of semaglutide on liver health in the overall population of patients in SELECT with overweight or obesity and established ASCVD. Liver enzymes, ALT, AST and GGT, as well as FLI, an algorithm based on BMI, waist circumference, triglycerides and GGT predictive of hepatic steatosis^39^, were assessed over 104 weeks.

Blood samples were collected at patient enrollment or randomization, then subsequently at weeks 20, 52, 104, 156, 208 and the end of the patient’s treatment phase. In certain European countries, additional blood tests were undertaken between weeks 4 and 16 given mandated safety requirements (Austria, Belgium, Czech Republic, Denmark, Finland, France, Germany, Greece, Hungary, Republic of Ireland, Italy, Latvia, the Netherlands, Norway, Poland, Portugal, Romania, Spain, Sweden and the UK).

### Statistical analysis

The statistical analyses were based on the intention-to-treat principle using data from the in-trial period and included all randomized patients irrespective of adherence to semaglutide or placebo or changes to background medications. Time-to-event endpoints were analyzed using a Cox proportional hazards model with treatment group (semaglutide, placebo) as fixed factors together with the two-sided 95% CI and two-sided *P* values; subgroup analyses were based on the same model by adding an interaction between treatment group and the specific subgroup as a factor. Continuous endpoints were analyzed using an analysis of covariance model with treatment as a fixed factor and baseline value of the endpoint as a covariate, where the ratio to baseline and the corresponding baseline value were log-transformed before the analysis. Missing data at week 104 were imputed using a multiple imputation model, conducted separately for each treatment arm and including baseline value as a covariate, and fitted to patients having an observed datapoint (irrespective of adherence to randomized treatment) at week 104. The fit model was used to impute values for all patients with missing data at week 104 to create 500 complete datasets. Rubin’s rules were used to combine the results. CIs were not adjusted for multiplicity; therefore, they should not be used to infer definitive treatment effects. All statistical analyses were performed using the SAS software v.9.4 (SAS Institute).

### Reporting summary

Further information on research design is available in the Nature Portfolio Reporting Summary linked to this article.

## Online content

Any methods, additional references, Nature Portfolio reporting summaries, source data, extended data, supplementary information, acknowledgements, peer review information; details of author contributions and competing interests; and statements of data and code availability are available at 10.1038/s41591-026-04281-1.

## Supplementary information

Supplementary Information Full list of SELECT Consortia investigators.

Reporting Summary

## Source data

Source Data Fig. 1 Statistical source data.

Source Data Fig. 2 Statistical source data.

Source Data Fig. 3 Statistical source data.

Source Data Extended Fig.1 Statistical source data.

## Figures and Tables

**Extended Data Table 1:**
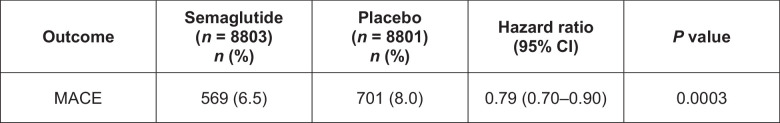
Time to first MACE Cox regression analysis with change in body weight as a time-dependent covariate

Analysis is based on a Cox proportional hazards model with treatment (semaglutide, placebo) as a fixed factor, baseline body weight as a covariate and change in body weight after randomization as a time-dependent covariate. Missing measurements at planned visits were imputed with LOCF.

CI, confidence interval; LOCF, last observation carried forward; MACE, major adverse cardiovascular event.

**Extended Data Table 2:**
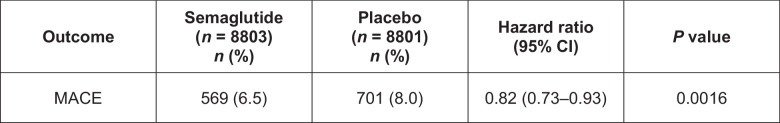
Time to first MACE Cox regression analysis with change in HbA_1c_ as a time-dependent covariate

Analysis is based on a Cox proportional hazards model with treatment (semaglutide, placebo) as a fixed factor, baseline HbA_1c_ as a covariate and change in HbA_1c_ after randomization as a time-dependent covariate. Missing measurements at planned visits were imputed with LOCF.

CI, confidence interval; HbA_1c_, glycated hemoglobin; LOCF, last observation carried forward; MACE, major adverse cardiovascular event.

## Data Availability

Authorized researchers can request access to clinical trial data by submitting a research proposal for review and approval by Novo Nordisk and an internal independent review panel. Requests are usually considered after the research is finished and the main results have been published. If the research supports a regulatory application, requests will be considered after the product and its intended use are approved in both the European Union and the USA. Participant clinical data will be anonymized, after an approved internal process, before they are shared with external third parties. For details on how to request access to clinical data, visit novonordisk-trials.com or contact Maria Quiroga (mdzp@novonordisk.com). Source data are provided with this paper.

## References

[1] Tacke, F. et al. EASL-EASD-EASO Clinical Practice Guidelines on the management of metabolic dysfunction-associated steatotic liver disease (MASLD). *J. Hepatol.* **81**, 492–542 (2024). 38851997 10.1016/j.jhep.2024.04.031

[2] Chalasani, N. et al. The diagnosis and management of nonalcoholic fatty liver disease: practice guidance from the American Association for the Study of Liver Diseases. *Hepatology* **67**, 328–357 (2018). 28714183 10.1002/hep.29367

[3] Cariou, B. The metabolic triad of non-alcoholic fatty liver disease, visceral adiposity and type 2 diabetes: implications for treatment. *Diabetes Obes. Metab.* **24**, 15–27 (2022). 35014161 10.1111/dom.14651

[4] Le, M. H. et al. Global incidence of non-alcoholic fatty liver disease: a systematic review and meta-analysis of 63 studies and 1,201,807 persons. *J. Hepatol.* **79**, 287–295 (2023). 37040843 10.1016/j.jhep.2023.03.040

[5] Quek, J. et al. Global prevalence of non-alcoholic fatty liver disease and non-alcoholic steatohepatitis in the overweight and obese population: a systematic review and meta-analysis. *Lancet Gastroenterol. Hepatol.* **8**, 20–30 (2023). 36400097 10.1016/S2468-1253(22)00317-X

[6] Mantovani, A. et al. Non-alcoholic fatty liver disease and risk of fatal and non-fatal cardiovascular events: an updated systematic review and meta-analysis. *Lancet Gastroenterol. Hepatol.* **6**, 903–913 (2021). 34555346 10.1016/S2468-1253(21)00308-3

[7] Meyersohn, N. M. et al. Association of hepatic steatosis with major adverse cardiovascular events, independent of coronary artery disease. *Clin. Gastroenterol. Hepatol.* **19**, 1480–1488 (2021). 32707340 10.1016/j.cgh.2020.07.030 PMC7855524

[8] Baratta, F. et al. Nonalcoholic fatty liver disease and fibrosis associated with increased risk of cardiovascular events in a prospective study. *Clin. Gastroenterol. Hepatol.* **18**, 2324–2331 (2020). 31887443 10.1016/j.cgh.2019.12.026

[9] Rinella, M. E. et al. AASLD Practice Guidance on the clinical assessment and management of nonalcoholic fatty liver disease. *Hepatology* **77**, 1797–1835 (2023). 36727674 10.1097/HEP.0000000000000323 PMC10735173

[10] Shah, A. G. et al. Comparison of noninvasive markers of fibrosis in patients with nonalcoholic fatty liver disease. *Clin. Gastroenterol. Hepatol.* **7**, 1104–1112 (2009). 19523535 10.1016/j.cgh.2009.05.033 PMC3079239

[11] McPherson, S. et al. Age as a confounding factor for the accurate non-invasive diagnosis of advanced NAFLD fibrosis. *Am. J. Gastroenterol.* **112**, 740–751 (2017). 27725647 10.1038/ajg.2016.453 PMC5418560

[12] Lincoff, A. M. et al. Semaglutide and cardiovascular outcomes in obesity without diabetes. *N. Engl. J. Med.* **389**, 2221–2232 (2023). 37952131 10.1056/NEJMoa2307563

[13] Sanyal, A. J. et al. Phase 3 trial of semaglutide in metabolic dysfunction-associated steatohepatitis. *N. Engl. J. Med.* **392**, 2089–2099 (2025). 40305708 10.1056/NEJMoa2413258

[14] Henning, R. J. Obesity and obesity-induced inflammatory disease contribute to atherosclerosis: a review of the pathophysiology and treatment of obesity. *Am. J. Cardiovasc. Dis.* **11**, 504–529 (2021). 34548951 PMC8449192

[15] Laakso, M. & Kuusisto, J. Insulin resistance and hyperglycaemia in cardiovascular disease development. *Nat. Rev. Endocrinol.* **10**, 293–302 (2014). 24663222 10.1038/nrendo.2014.29

[16] Danesh, J., Collins, R., Appleby, P. & Peto, R. Association of fibrinogen, C-reactive protein, albumin, or leukocyte count with coronary heart disease: meta-analyses of prospective studies. *JAMA* **279**, 1477–1482 (1998). 9600484 10.1001/jama.279.18.1477

[17] Eguchi, Y. et al. Visceral fat accumulation and insulin resistance are important factors in nonalcoholic fatty liver disease. *J. Gastroenterol.* **41**, 462–469 (2006). 16799888 10.1007/s00535-006-1790-5

[18] Azzu, V., Vacca, M., Virtue, S., Allison, M. & Vidal-Puig, A. Adipose tissue-liver cross talk in the control of whole-body metabolism: implications in nonalcoholic fatty liver disease. *Gastroenterology* **158**, 1899–1912 (2020). 32061598 10.1053/j.gastro.2019.12.054

[19] Cariou, B., Byrne, C. D., Loomba, R. & Sanyal, A. J. Nonalcoholic fatty liver disease as a metabolic disease in humans: a literature review. *Diabetes Obes. Metab.* **23**, 1069–1083 (2021). 33464677 10.1111/dom.14322 PMC8248154

[20] Liao, C., Liang, X., Zhang, X. & Li, Y. The effects of GLP-1 receptor agonists on visceral fat and liver ectopic fat in an adult population with or without diabetes and nonalcoholic fatty liver disease: a systematic review and meta-analysis. *PLoS ONE* **18**, e0289616 (2023). 37616255 10.1371/journal.pone.0289616 PMC10449217

[21] Nevola, R. et al. GLP-1 receptor agonists in non-alcoholic fatty liver disease: current evidence and future perspectives. *Int. J. Mol. Sci.* **24**, 1703 (2023). 36675217 10.3390/ijms24021703 PMC9865319

[22] Masson, W. et al. Anti-inflammatory effect of semaglutide: updated systematic review and meta-analysis. *Front. Cardiovasc. Med.* **11**, 1379189 (2024). 39055657 10.3389/fcvm.2024.1379189 PMC11270812

[23] Hu, Q. et al. Semaglutide ameliorates hepatocyte steatosis in a cell co-culture system by downregulating the IRE1α-XBP1-C/EBPα signaling pathway in macrophages. *Pharmacology* **110**, 26–35 (2025). 39089233 10.1159/000540654

[24] Pan, X. et al. Semaglutide ameliorates obesity-induced cardiac inflammation and oxidative stress mediated via reduction of neutrophil Cxcl2, S100a8, and S100a9 expression. *Mol. Cell. Biochem.* **479**, 1133–1147 (2024). 37318712 10.1007/s11010-023-04784-2

[25] Iacobellis, G. & Villasante Fricke, A. C. Effects of semaglutide versus dulaglutide on epicardial fat thickness in subjects with type 2 diabetes and obesity. *J. Endocr. Soc.* **4**, bvz042 (2020). 32190806 10.1210/jendso/bvz042 PMC7069837

[26] García-Vega, D. et al. Semaglutide modulates prothrombotic and atherosclerotic mechanisms, associated with epicardial fat, neutrophils and endothelial cells network. *Cardiovasc. Diabetol.* **23**, 1 (2024). 38172989 10.1186/s12933-023-02096-9 PMC10765851

[27] Al Refaie, A. et al. Adiponectin may play a crucial role in the metabolic effects of GLP-1RAs treatment in patients with type 2 diabetes mellitus: a preliminary longitudinal study. *Endocrine* **87**, 951–958 (2025). 39521749 10.1007/s12020-024-04085-8

[28] Koutoukidis, D. A. et al. The effect of the magnitude of weight loss on non-alcoholic fatty liver disease: a systematic review and meta-analysis. *Metabolism* **115**, 154455 (2021). 33259835 10.1016/j.metabol.2020.154455

[29] Flint, A. et al. Randomised clinical trial: semaglutide versus placebo reduced liver steatosis but not liver stiffness in subjects with non-alcoholic fatty liver disease assessed by magnetic resonance imaging. *Aliment. Pharmacol. Ther.* **54**, 1150–1161 (2021). 34570916 10.1111/apt.16608 PMC9292692

[30] Vilar-Gomez, E. et al. Weight loss through lifestyle modification significantly reduces features of nonalcoholic steatohepatitis. *Gastroenterology* **149**, 367–378 (2015). 25865049 10.1053/j.gastro.2015.04.005

[31] Newsome, P. N. et al. A placebo-controlled trial of subcutaneous semaglutide in nonalcoholic steatohepatitis. *N. Engl. J. Med.* **384**, 1113–1124 (2021). 33185364 10.1056/NEJMoa2028395

[32] Verrastro, O. et al. Bariatric-metabolic surgery versus lifestyle intervention plus best medical care in non-alcoholic steatohepatitis (BRAVES): a multicentre, open-label, randomised trial. *Lancet* **401**, 1786–1797 (2023). 37088093 10.1016/S0140-6736(23)00634-7

[33] Hwang, J. et al. Bariatric intervention improves metabolic dysfunction-associated steatohepatitis in patients with obesity: a systematic review and meta-analysis. *Clin. Mol. Hepatol.* **30**, 561–576 (2024). 38830642 10.3350/cmh.2023.0384 PMC11261233

[34] Kahn, S. E. et al. Effect of semaglutide on regression and progression of glycemia in people with overweight or obesity but without diabetes in the SELECT trial. *Diabetes Care* **47**, 1350–1359 (2024). 38907683 10.2337/dc24-0491 PMC11282386

[35] Pradhan, R. et al. Glucagon-like peptide 1 receptor agonists and sodium-glucose cotransporter 2 inhibitors and the prevention of cirrhosis among patients with type 2 diabetes. *Diabetes Care* **48**, 444–454 (2025). 39774820 10.2337/dc24-1903

[36] Patel, K. & Sebastiani, G. Limitations of non-invasive tests for assessment of liver fibrosis. *JHEP Rep.* **2**, 100067 (2020). 32118201 10.1016/j.jhepr.2020.100067 PMC7047178

[37] Ryan, D. H. et al. Semaglutide effects on cardiovascular outcomes in people with overweight or obesity (SELECT) rationale and design. *Am. Heart J.* **229**, 61–69 (2020). 32916609 10.1016/j.ahj.2020.07.008

[38] Lingvay, I. et al. Semaglutide for cardiovascular event reduction in people with overweight or obesity: SELECT study baseline characteristics. *Obesity* **31**, 111–122 (2023). 36502289 10.1002/oby.23621 PMC10107832

[39] Bedogni, G. et al. The fatty liver index: a simple and accurate predictor of hepatic steatosis in the general population. *BMC Gastroenterol.* **6**, 33 (2006). 17081293 10.1186/1471-230X-6-33 PMC1636651

