# Supplementary material for: Semaglutide on liver fibrosis and heart outcomes in patients at high risk of liver fibrosis: a prespecified analysis of the SELECT randomized trial
Source: Nat Med. 2026 Apr 2;32(5):1686–93. doi: 10.1038/s41591-026-04281-1 (PMC13190271; doi:10.1038/s41591-026-04281-1)
Supplement: Supplementary file 1 — Full list of SELECT Consortia investigators. [file 41591_2026_4281_MOESM1_ESM.pdf]

nature medicine

Supplementary information https://doi.org/10.1038/s41591-026-04281-1

Semaglutide on liver fibrosis and heart outcomes in patients at high risk of liver fibrosis: a prespecified analysis of the SELECT randomized trial

In the format provided by the authors and unedited

Supplemental Online Content: Nonauthor Collaborators
*First name, last name, and suffix (if applicable) are required and will appear in PubMed.

*Group Name(s): SELECT Trial Investigators							
***First Name and Middle Initial(s)**	***Last Name**	***Suffix (eg, Jr, III)**	Academic Degrees	Institution	Location (city, state/province, country)	Role or Contribution, eg, chair, principal investigator	Group (if more than 1 Group listed in the byline) and/or Subgroup (eg, Steering Committee)
Djamel E.	Nibouche			CHU - Hussein dey Cardiology department Nafissa Hamoud	Algeria	Principal investigator	
Mohamed	Chettibi			CHU Issad Hassani, Beni Messous, Cardiology department	Algeria	Principal investigator	
Naima	Hammoudi			Centre National De la Médecine du Sport	Algeria	Principal investigator	
Alejandro	Porto			Glenny Corp. S.A	Argentina	Principal investigator	
Alejandra	Oviedo			Medical Center of Diabetes and Nutrition	Argentina	Principal investigator	
Hugo D.	Sanabria			Instituto Cardiovascular Buenos Aires	Argentina	Principal investigator	
Marcelo	Casas			Clínica Adventista Belgrano	Argentina	Principal investigator	
Eduardo	Farias			Instituto de Cardiología de Corrientes	Argentina	Principal investigator	
Ernesto	Duronto			Fundacion Favaloro	Argentina	Principal investigator	
Carolina	Chacon			Sanatorio Plaza	Argentina	Principal investigator	
Adriana	Villarino			Instituto Médico DAMIC	Argentina	Principal investigator	
Javier M.	Farias			Sanatorio Güemes	Argentina	Principal investigator	
Cesar J.	Zaidman			Centro de Investigación y Prevención Cardiovascular	Argentina	Principal investigator	
Claire	Morbey			Australian institute of metabolism	Australia	Principal investigator	
David M.	Colquhoun			Core Research Centre	Australia	Principal investigator	
Elif I.	Ekinci			Austin Health, Metabolic Disorders Centre	Australia	Principal investigator	
Melissa	Leung			Liverpool Hospital	Australia	Principal investigator	
Maged	William			Gosford Hospital	Australia	Principal investigator	
Karam	Kostner			Cholesterol Care Australia	Australia	Principal investigator	
Mitra	Shirazi			Royal Adelaide Hospital Cardiovascular Clinical Trials	Australia	Principal investigator	

Supplemental Online Content: Nonauthor Collaborators
*First name, last name, and suffix (if applicable) are required and will appear in PubMed.

***First Name and Middle Initial(s)**	***Last Name**	***Suffix (eg, Jr, III)**	Academic Degrees	Institution	Location (city, state/province, country)	Role or Contribution, eg, chair, principal investigator	Group (if more than 1 Group listed in the byline) and/or Subgroup (eg, Steering Committee)
David	Cross			Advara HeartCare	Australia	Principal investigator	
John	Amerena			Geelong Cardiology Research Unit	Australia	Principal investigator	
Peter	Purnell			HeartCare WA (Joondalup)	Australia	Principal investigator	
Margaret	Arstall			Lyell McEwin Hospital	Australia	Principal investigator	
Walter P.	Abhayaratna			The Canberra Hospital_Garran	Australia	Principal investigator	
Hermann	Toplak			Universitätsklinik für Innere Medizin	Austria	Principal investigator	
Rudolf	Prager			Klinik Hietzing	Austria	Principal investigator	
Bernhard	Ludvik			Klinik Landstraße	Austria	Principal investigator	
Alexandra	Kautzky-Willer			Universitätsklinikum AKH Wien	Austria	Principal investigator	
Ursula	Hanusch			Ordination Dr. Hanusch	Austria	Principal investigator	
Elmar	Aigner			LKH Salzburg Universitätsklinikum	Austria	Principal investigator	
Susanne	Kaser			Univ.-Klinik für Innere Medizin I	Austria	Principal investigator	
Michel	De Pauw			UZ Gent_Gent_4	Belgium	Principal investigator	
Philippe	Vanduynhoven			Algemeen Stedelijk Ziekenhuis - Aalst - Interventional Cardiology	Belgium	Principal investigator	
Philippe	Van De Borne			CUB Hôpital Erasme_Brussels_1	Belgium	Principal investigator	
Chris	Vercammen			Imeldaziekenhuis - Bonheiden - Department of Endocrinology	Belgium	Principal investigator	
Jan	Verwerft			Jessa Ziekenhuis - Hasselt - Cardiology	Belgium	Principal investigator	
Mathias	Vrolix			Ziekenhuis Oost-Limburg AV - Cardiology	Belgium	Principal investigator	
Luiz A.	Turatti			CPQuali Pesquisa Clínica Ltda	Brazil	Principal investigator	
José F.	Saraiva			Instituto de Pesquisa Clínica de Campinas	Brazil	Principal investigator	
Cintia	Cercato			Departamento de Gastroenterologia - HCFMUSP	Brazil	Principal investigator	
Adrian P.	Kormann			AngioCor Blumenau	Brazil	Principal investigator	
Silmara O.	Leite			Cline Research Center	Brazil	Principal investigator	
Paulo	Rossi			Núcleo de Pesquisa Clínica S/S	Brazil	Principal investigator	

Supplemental Online Content: Nonauthor Collaborators
*First name, last name, and suffix (if applicable) are required and will appear in PubMed.

*First Name and Middle Initial(s)	*Last Name	*Suffix (eg, Jr, III)	Academic Degrees	Institution	Location (city, state/province, country)	Role or Contribution, eg, chair, principal investigator	Group (if more than 1 Group listed in the byline) and/or Subgroup (eg, Steering Committee)
Flávia B.	Arantes			Eurolatino Medical Research Center	Brazil	Principal investigator	
Lilia N.	Maia			CIP Centro Integrado de Pesquisas do Hospital de Base	Brazil	Principal investigator	
Sergio E.	Kaiser			Instituto Brasil de Pesquisa Clinica	Brazil	Principal investigator	
Alberto G.	Fonseca			Hospital do Coração do Brasil	Brazil	Principal investigator	
Hugo R.	Kurtz Lisboa			Hospital São Vicente de Paulo	Brazil	Principal investigator	
Denise R.	Franco			CPCLIN - Centro de Pesquisas Clínicas	Brazil	Principal investigator	
José C.	Nicolau			Instituto do Coração - HCFMUSP	Brazil	Principal investigator	
Pedro S.	Farsky			Instituto Dante Pazzanese de Cardiologia	Brazil	Principal investigator	
Luiz E.	Ritt			Cárdio Pulmonar da Bahia	Brazil	Principal investigator	
Assen	Goudev			"UMHAT "Tsaritsa Yoanna-ISUL"" EAD, Clinic of Cardiology	Bulgaria	Principal investigator	
Dobrin	Vassilev			"UMHAT "Aleksandrovska" EAD, Cardiology clinic	Bulgaria	Principal investigator	
Nikolay	Runev			"Diagnostic - Consulting Center Aleksandrovska" EOOD	Bulgaria	Principal investigator	
Borislav	Georgiev			"MHAT "NCH" EAD, Clinic of Cardiology, Depart. of Cardiology	Bulgaria	Principal investigator	
Tzvetana	Katova			"MHAT "NCH" EAD, Cardiology clinic, Noninvasive diag. dep.	Bulgaria	Principal investigator	
Ivo	Petrov			"Acibadem City Clinic UMHAT? EOOD, Sofia, Cardiology clinic	Bulgaria	Principal investigator	
Valeri	Gelev			?Acibadem City Clinic MHAT Tokuda? EAD,Cardiology department	Bulgaria	Principal investigator	
Maria	Milanova			"UMHATEM N.I. Pirogov" EAD	Bulgaria	Principal investigator	
Arman	Postadzhiyan			"UMHAT "Sveta Anna" Sofia" AD, Clinic of Cardiology	Bulgaria	Principal investigator	

Supplemental Online Content: Nonauthor Collaborators
*First name, last name, and suffix (if applicable) are required and will appear in PubMed.

***First Name and Middle Initial(s)**	***Last Name**	***Suffix (eg, Jr, III)**	Academic Degrees	Institution	Location (city, state/province, country)	Role or Contribution, eg, chair, principal investigator	Group (if more than 1 Group listed in the byline) and/or Subgroup (eg, Steering Committee)
Mariya	Tokmakova			"UMHAT "Sveti Georgi" EAD	Bulgaria	Principal investigator	
Yoto	Yotov			"Diagnostic - Consulting Center "Ekvita"" EOOD	Bulgaria	Principal investigator	
Snezhanka	Tisheva-Gospodinova			"UMBAL-Dr. Georgi Stranski" EAD, First Clinic of cardiology	Bulgaria	Principal investigator	
Dotska A.	Minkova			Medical centre Razgrad OOD	Bulgaria	Principal investigator	
Zhulieta R.	Prakova-Teneva			Medical Centre Diamedical-2013	Bulgaria	Principal investigator	
Svetla P.	Vasileva			MHAT Lukovit, Department of Internal Diseases	Bulgaria	Principal investigator	
Alan	Egan			Synergy Wellness Clinic	Canada	Principal investigator	
George M.	Tsoukas			Applied Med Inf Res	Canada	Principal investigator	
Subodh	Verma			North York Diagn & Cardiac Ctr	Canada	Principal investigator	
Ronald	Bourgeois			G.A. Research Associates Ltd.	Canada	Principal investigator	
Pierre	Filteau			Ctr Méd. et pro d l'Ost d port	Canada	Principal investigator	
Ram	Vijayaraghavan			HHI Research Inc.	Canada	Principal investigator	
Michael	Hartleib			Kawartha Cardiology Clinical Trials	Canada	Principal investigator	
Tamara	Spaic			St. Joseph's Health Care	Canada	Principal investigator	
John	Stewart			Medical Associates of Port Perry	Canada	Principal investigator	
Amritanshu- S.	Pandey			Cambridge Cardiac Care Centre	Canada	Principal investigator	
Remi	Rabasa-Lhoret			IRCM	Canada	Principal investigator	
Francois	St-Maurice			ViaCar Recherche Clinique Inc	Canada	Principal investigator	
James Y.	Cha			Dr. James Cha	Canada	Principal investigator	
Sue	Pedersen			C-endo Diab Endo Clin Calgery	Canada	Principal investigator	
Roy	Allison			The Port Arthur Clinic Research Program	Canada	Principal investigator	
Paul	Poirier			Institut universitaire de cardiologie	Canada	Principal investigator	
Marie-France	Langlois			Centre de recherché du CHUS	Canada	Principal investigator	
David	Lau			University of Calgary	Canada	Principal investigator	
Yves	Robitaille			Centre de santé et de services	Canada	Principal investigator	

*First name, last name, and suffix (if applicable) are required and will appear in PubMed.

***First Name and Middle Initial(s)**	***Last Name**	***Suffix (eg, Jr, III)**	Academic Degrees	Institution	Location (city, state/province, country)	Role or Contribution, eg, chair, principal investigator	Group (if more than 1 Group listed in the byline) and/or Subgroup (eg, Steering Committee)
John	Weisnagel			Clin des Mal Lipid de Quebec	Canada	Principal investigator	
Christian	Constance			Clinique Sante Cardio MC	Canada	Principal investigator	
Sudip	Datta			Manna Research Stoney Creek	Canada	Principal investigator	
Naveen	Garg			Manna Research Inc.	Canada	Principal investigator	
Tim	Salter			Manna Research Inc. (Burlington N)	Canada	Principal investigator	
Noah	Vale			Manna Research Inc._Toronto	Canada	Principal investigator	
Gerald	Vallieres			Manna Research Quebec	Canada	Principal investigator	
Karina	Peters			Manna Research Ottawa	Canada	Principal investigator	
Guy	Tellier			Manna Research Mirabel	Canada	Principal investigator	
Paul	Poirier			Institut universitaire de cardiologie	Canada	Principal investigator	
Amritanshu- S.	Pandey			Cambridge Cardiac Care Centre	Canada	Principal investigator	
Rodrigo	Botero Lopez			Rodrigo Botero S.A.S	Colombia	Principal investigator	
Miguel A.	Urina Triana			Fundacion del Caribe para la Investigacion Biomedica-BIOS	Colombia	Principal investigator	
Alberto J.	Cadena Bonfanti			Clinica de la Costa	Colombia	Principal investigator	
Jose L.	Accini Mendoza			IPS Centro Cientifico Asistencial Jose Luis Accini SAS	Colombia	Principal investigator	
Jaime A.	Rodriguez Plazas			Unidad de Estudios Clínicos de la Fundación Cardiovascular de Colombia	Colombia	Principal investigator	
Carlos A.	Martinez Cano			CardiovidCentro Cardiovascular Colombiano ClinicaSantaMaria	Colombia	Principal investigator	
Davor	Milicic			KBC Zagreb, Klinika za bolesti srca i krvnih zila	Croatia	Principal investigator	
Silvija	Canecki Varzic			Klinicki bolnicki centar Osijek	Croatia	Principal investigator	
Hrvoje	Budincevic			Klinicka bolnica Sveti Duh	Croatia	Principal investigator	
Diana	Delic-Brkljacic			KBC "Sestre milosrdnice", Kardiologija	Croatia	Principal investigator	

Supplemental Online Content: Nonauthor Collaborators
*First name, last name, and suffix (if applicable) are required and will appear in PubMed.

*First Name and Middle Initial(s)	*Last Name	*Suffix (eg, Jr, III)	Academic Degrees	Institution	Location (city, state/province, country)	Role or Contribution, eg, chair, principal investigator	Group (if more than 1 Group listed in the byline) and/or Subgroup (eg, Steering Committee)
Alen	Ruzic			KBC Rijeka, Zavod za kardiomiopatije	Croatia	Principal investigator	
Ivan	Kruljac			Poliklinika Solmed	Croatia	Principal investigator	
Krunoslav	Fuckar			Specijalna bolnica Krapinske Toplice - Kardiologija	Croatia	Principal investigator	
Ales	Linhart			Fakultni poliklinika	Czechia	Principal investigator	
Jan	Malecha			Ordinace pro choroby srdce	Czechia	Principal investigator	
Ondrej	Cermak			Nemocnice Slany Kardiologie	Czechia	Principal investigator	
Pavel	Koleckar			Svitavska nemocnice Kardiologie	Czechia	Principal investigator	
Zdenek	Klimsa			Nemocnice Jihlava Kardiologie	Czechia	Principal investigator	
Ladislav	Kuchar			Vseobecna kardiologicka ambulance	Czechia	Principal investigator	
Emilia	Malicherova			ResTrial	Czechia	Principal investigator	
Pavel	Koleckar			Policska nemocnice	Czechia	Principal investigator	
Josef	Stasek			Fakultni nemocnice Hradec Kralove_Hradec Kralove_0	Czechia	Principal investigator	
Martin	Haluzik			Endocare	Czechia	Principal investigator	
Alica	Vesela			Edumed Broumov	Czechia	Principal investigator	
Michael	Mæng			Aarhus Universitetshospital, Skejby Hjertesygdomme	Denmark	Principal investigator	
Jens D.	Hove			Hvidovre Hospital Hjertemedicinsk	Denmark	Principal investigator	
Kenneth	Egstrup			Kardiologisk Odense & Svendborg	Denmark	Principal investigator	
Svend E.	Jensen			Aalborg Universitetshospital Kardiologisk Afdeling	Denmark	Principal investigator	
Gunnar	Gislason			Herlev og Gentofte Hospital	Denmark	Principal investigator	
Morten	Bôttcher			Regionshospitalet Gødstrup, Hjertesygdomme	Denmark	Principal investigator	
Mikko	Pietilä			TYKS Sydänkeskus	Finland	Principal investigator	
Erkki	Ilveskoski			TAYS Sydänsairaala	Finland	Principal investigator	
Timo	Lakka			Health Step Finland Oy	Finland	Principal investigator	

*First name, last name, and suffix (if applicable) are required and will appear in PubMed.

*First Name and Middle Initial(s)	*Last Name	*Suffix (eg, Jr, III)	Academic Degrees	Institution	Location (city, state/province, country)	Role or Contribution, eg, chair, principal investigator	Group (if more than 1 Group listed in the byline) and/or Subgroup (eg, Steering Committee)
Petteri	Ahtiainen			StudyCor	Finland	Principal investigator	
Olavi	Ukkola			OYS Sisätautien tutkimusyksikkö	Finland	Principal investigator	
Leo	Niskanen			Päijät-Häme Central Hospital	Finland	Principal investigator	
Tiina	Keski-Opas			Seinäjoen keskussairaala	Finland	Principal investigator	
François	Schiele			CHRU Besançon	France	Principal investigator	
Dominique	Stephan			Nouvel Hôpital Civil	France	Principal investigator	
Catherine	Petit			Centre Hospitalier Sud Francilien	France	Principal investigator	
Mathieu	Kerneis			CHU PITIE-SALPETRIERE	France	Principal investigator	
Bertrand	Cariou			HÔPITAL NORD LAËNNEC- SERVICE D'ENDOCRINOLOGIE	France	Principal investigator	
Gabriel	Steg			Hôpital Bichat - Claude Bernard - Cardio	France	Principal investigator	
Bruno	Verges			CHU DU BOCAGE	France	Principal investigator	
Jean-Sébastien	Hulot			Hôpital Européen Georges Pompidou	France	Principal investigator	
Thibaud	Demicheli			CH Louis Pasteur	France	Principal investigator	
Jean-Francois	Thuan			Centre Hospitalier Général de NARBONNE	France	Principal investigator	
Gilles	Barone Rochette			CHU de Grenoble - Alpes	France	Principal investigator	
Blandine	Gatta-Cherifi			HOPITAL HAUT LEVEQUE-PESSAC	France	Principal investigator	
Pierre	Serusclat			Groupe Hospitalier Mutualiste des Portes du Sud	France	Principal investigator	
Ayham	Al-Zoebi			Al-Zoebi	Germany	Principal investigator	
Karl-Friedrich	Appel			Appel	Germany	Principal investigator	
Kristin	Kreutzmann			Hausaerztlich-Kardiologisches MVZ Am Felsenkeller GmbH	Germany	Principal investigator	
Julia	Chevts			Chevts	Germany	Principal investigator	
Ralf J.	Denger			Denger, Friedrichsthal	Germany	Principal investigator	
Holger	Eggebrecht			MVZ CCB Frankfurt Und Main-Taunus GbR	Germany	Principal investigator	

*First name, last name, and suffix (if applicable) are required and will appear in PubMed.

*First Name and Middle Initial(s)	*Last Name	*Suffix (eg, Jr, III)	Academic Degrees	Institution	Location (city, state/province, country)	Role or Contribution, eg, chair, principal investigator	Group (if more than 1 Group listed in the byline) and/or Subgroup (eg, Steering Committee)
Charlotte	Von Engelhardt			Klinische Forschung Schwerin	Germany	Principal investigator	
Andreas	Hagenow			Zentrum fuer klinische Studien Suedbrandenburg GmbH	Germany	Principal investigator	
Peter	Heymer			Klinische Forschung Dresden	Germany	Principal investigator	
Stephan	Jacob			Jacob, Villingen-Schwenningen	Germany	Principal investigator	
Wolfgang	Jungmair			Kardiologische Praxis	Germany	Principal investigator	
Gerd	Kahrmann			Kahrmann	Germany	Principal investigator	
Hans-Peter	Kempe			Die Praxis am Ludwigsplatz	Germany	Principal investigator	
Candy	Von Münchhausen			Klinische Forschung Berlin (kfgn)	Germany	Principal investigator	
Michael	Lehrke			Universitaetsklinikum Aachen AöR	Germany	Principal investigator	
Jan	Wagner			Klinische Forschung Hannover	Germany	Principal investigator	
Michael	Froer			Klinische Forschung Hamburg	Germany	Principal investigator	
Joachim	Sauter			Sauter, Wangen	Germany	Principal investigator	
Sebastian	Meyhöfer			Universitätsklinikum Schleswig-Holstein	Germany	Principal investigator	
Helga	Zeller			InnoDiab Forschung GmbH	Germany	Principal investigator	
Alexander	Segner			Segner, St. Ingbert	Germany	Principal investigator	
Hans-Detlev	Stahl			AmBeNet GmbH	Germany	Principal investigator	
Joerg	Steindorf			Steindorf, Schkeuditz	Germany	Principal investigator	
Kerstin	Sturm			Emovis GmbH	Germany	Principal investigator	
Thomas	Schaum			RED-Institut für medizinische Studien und Fortbildung GmbH	Germany	Principal investigator	
Ulrich	Wendisch			Wendisch/Dahl Hamburg	Germany	Principal investigator	
Evangelos	Liberopoulos			University General Hospital of Ioannina,Internal Medicine	Greece	Principal investigator	
Alexandra	Bargiota			Univ Gen Hospital Larisa, Endocrinology & Metabolic Disease	Greece	Principal investigator	
Konstantinos	Tziomalos			AHEPA General University Hospital	Greece	Principal investigator	

Supplemental Online Content: Nonauthor Collaborators
*First name, last name, and suffix (if applicable) are required and will appear in PubMed.

*First Name and Middle Initial(s)	*Last Name	*Suffix (eg, Jr, III)	Academic Degrees	Institution	Location (city, state/province, country)	Role or Contribution, eg, chair, principal investigator	Group (if more than 1 Group listed in the byline) and/or Subgroup (eg, Steering Committee)
Vaia	Lambadiari			University Hospital of Athens ATTIKON	Greece	Principal investigator	
Emmanouil	Pagkalos			"Thermi" Private Hosital	Greece	Principal investigator	
Alexander	Kokkinos			"Laiko" General Hospital of Athens	Greece	Principal investigator	
Georgia	Argyrakopoulou			Iatriko Athinon (Athens Medical Canter)	Greece	Principal investigator	
Gerasimos	Siasos			Genl Hospital of Athens Ippokrateio,A? Cardiology Univ dpt	Greece	Principal investigator	
Sotirios	Patsilinakos			Konstantopouleio G.H. of Athens, "Agia Olga"	Greece	Principal investigator	
Leonidas	Lanaras			General Hospital of Lamia	Greece	Principal investigator	
Athanasios	Kartalis			General Hospital of Chios ?Skilitsio?	Greece	Principal investigator	
George	Ntaios			Univ Gen Hospital Larisa, Internal Medicine Clinic	Greece	Principal investigator	
Kimon	Stamatelopoulos			Alexandra General Hospital, Therapeutic Clinic	Greece	Principal investigator	
Elias	Tsougos			"Hygeia? General Hospital of Athens	Greece	Principal investigator	
Dénes	Páll			Debreceni Egyetem Klinikai Központ Belgyógyászati Klinika	Hungary	Principal investigator	
Gyozo	Kocsis			Uno Medical Trials Eü. Szolgáltató és Kereskedelmi Kft.	Hungary	Principal investigator	
Róbert	Takács			Szegedi Tudomanyegyetem St Györgyi Albert Klinikai Központ	Hungary	Principal investigator	
Marietta	Baranyai			Markusovszky Egyetemi Oktatókórház	Hungary	Principal investigator	
Géza	Lupkovics			Léda Platán Magánklinika	Hungary	Principal investigator	
Annamária	Piros			Bugát Pál Kórház	Hungary	Principal investigator	

*First name, last name, and suffix (if applicable) are required and will appear in PubMed.

***First Name and Middle Initial(s)**	***Last Name**	***Suffix (eg, Jr, III)**	Academic Degrees	Institution	Location (city, state/province, country)	Role or Contribution, eg, chair, principal investigator	Group (if more than 1 Group listed in the byline) and/or Subgroup (eg, Steering Committee)
László	Könyves			Lausmed Kft.	Hungary	Principal investigator	
Upendra	Kaul			Batra Hospital and Medical Research Center	India	Principal investigator	
Sanjay	Mittal			Medanta-The Medicity	India	Principal investigator	
Sawhney	Jitendra Pal Singh			Sir Ganga Ram Hospital	India	Principal investigator	
Sandeep	Garg			Maulana Azad Medical College	India	Principal investigator	
Roy	Ambuj			All India Institute of Medical Sciences	India	Principal investigator	
Dheeraj	Gandotra			BL Kapur Super Specialty Hospital	India	Principal investigator	
Nihal	Thomas			Christian Medical College Hospital, Vellore	India	Principal investigator	
Dhiman	Kahali			C K Birla Hospitals	India	Principal investigator	
Rajpal K.	Abhaichand			G Kuppuswamy Naidu Memorial Hospital	India	Principal investigator	
Johann	Christopher			Guru Nanak Care Hospitals	India	Principal investigator	
Shirish	Hiremath			Grant Medical Foundation	India	Principal investigator	
J	Kannan			Narayana Hrudayalya Institute of Cardiac Sciences	India	Principal investigator	
Rishi	Sethi			Gandhi Memorial Hospital- King George's Medical University	India	Principal investigator	
Manoj K.	Shah			Madras Medical Mission Hospital	India	Principal investigator	
Naik	Ajay			CIMS- Care Institute of Medical Sciences	India	Principal investigator	
Kerkar G.	Prafulla			Seth GS medical college and KEM Hospital	India	Principal investigator	
Kiron	Varghese			St John's Medical College and Hospital	India	Principal investigator	
Atul	Abhyankar			Shri B D Mehta Mahavir Heart Institute	India	Principal investigator	
Ajit	Bhagwat			Kamalnayan Bajaj Hospital	India	Principal investigator	

Supplemental Online Content: Nonauthor Collaborators
*First name, last name, and suffix (if applicable) are required and will appear in PubMed.

***First Name and Middle Initial(s)**	***Last Name**	***Suffix (eg, Jr, III)**	Academic Degrees	Institution	Location (city, state/province, country)	Role or Contribution, eg, chair, principal investigator	Group (if more than 1 Group listed in the byline) and/or Subgroup (eg, Steering Committee)
Gurpreet S.	Wander			Dayanand Medical College & Hospital	India	Principal investigator	
Arun K.	Chopra			Fortis Escorts Hospital	India	Principal investigator	
Krishna M.	Parvathareddy			Osmania General Hospital	India	Principal investigator	
Sandeep	Bansal			VMMC & Safdarjung Hospital	India	Principal investigator	
Ajay U.	Mahajan			Lokmanya Tilak Municipal Medical College & General Hospital	India	Principal investigator	
Ramesh B.	Pothineni			Ramesh Hospitals	India	Principal investigator	
Devang	Desai			Unicare heart institute and research centre	India	Principal investigator	
Vinod	Vijan			Vijan Cardiac & Critical Care	India	Principal investigator	
Devendra K.	Agarwal			SP Medical College & A.G.Hospital Bikaner	India	Principal investigator	
Mahesh	Fulwani			Shrikrishna Hrudayalaya and critical care centre	India	Principal investigator	
Jaspal	Arneja			Arneja Heart & Multispeciality Hospital	India	Principal investigator	
Piyush	Desai			Nirmal Hospital Pvt. Ltd.	India	Principal investigator	
Vincent	Maher			Tallaght Hospital - Cardiology	Ireland	Principal investigator	
David	Molony			Mallow Primary Healthcare Centre	Ireland	Principal investigator	
Kenneth	Mcdonald			The Heartbeat Trust	Ireland	Principal investigator	
Maria	Byrne			Mater Miscericordiae Hospital	Ireland	Principal investigator	
Donal	O'shea			Clinical Research Centre, St. Vincent's University Hospital,	Ireland	Principal investigator	
Seamus	Sreenan			Connolly Hospital	Ireland	Principal investigator	
Carel	Le Roux			St Vincent's Private Hospital	Ireland	Principal investigator	
Ofri	Mosenzon			Diabetes Unit Hadassah Ein Karem MC	Israel	Principal investigator	
Ronen	Durst			Hadassah MC - Cardio	Israel	Principal investigator	

*First name, last name, and suffix (if applicable) are required and will appear in PubMed.

***First Name and Middle Initial(s)**	***Last Name**	***Suffix (eg, Jr, III)**	Academic Degrees	Institution	Location (city, state/province, country)	Role or Contribution, eg, chair, principal investigator	Group (if more than 1 Group listed in the byline) and/or Subgroup (eg, Steering Committee)
Amir	Bashkin			Institute of Diabetes, Western Galilee MC Nahariya	Israel	Principal investigator	
Yael	Sofer			Institute of Endocrinology, metabolism and hypertension	Israel	Principal investigator	
Victor	Vishlitzky			Clinical Research Unit Meir Medical Center	Israel	Principal investigator	
Gabriella	Segal Lieberman			Endrocrinolgy Clinic - Sheba Medical Center	Israel	Principal investigator	
Michael	Shechter			Sheba Medica Center - Clinical Research Unit	Israel	Principal investigator	
Dror	Dicker			Rabin Medical Center - Hasharon Hospital, Dept. D	Israel	Principal investigator	
Taiba	Zornitzki			Kaplan MC	Israel	Principal investigator	
Idit	Liberty			Outpatient Diabetes Clinic Soroka MC	Israel	Principal investigator	
Naim	Shehadeh			Diabetes and obesity center of excellence, Rambam MC	Israel	Principal investigator	
Yaron	Arbel			Cardio Vascular Research Center Sourasky MC	Israel	Principal investigator	
Michael	Shechter			Sheba Medica Center - Clinical Research Unit	Israel	Principal investigator	
Silvio	Buscemi			Unità Funzionale di nutrizione clinica cod 58.01.3	Italy	Principal investigator	
Uberto	Pagotto			A. O. Universitaria S.ORSOLA-MALPIGHI - U. O. Endocrinologia	Italy	Principal investigator	
Roberto	Vettor			Azienda Ospedaliera di Padova Clin.Med.3	Italy	Principal investigator	
Ferruccio	Santini			Azienda Ospedaliero Universitaria Pisana Ospedale Cisanello	Italy	Principal investigator	
Geltrude	Mingrone			Policlinico Universitario AGemelli DH Patologie dell?Obesità	Italy	Principal investigator	

Supplemental Online Content: Nonauthor Collaborators
*First name, last name, and suffix (if applicable) are required and will appear in PubMed.

***First Name and Middle Initial(s)**	***Last Name**	***Suffix (eg, Jr, III)**	Academic Degrees	Institution	Location (city, state/province, country)	Role or Contribution, eg, chair, principal investigator	Group (if more than 1 Group listed in the byline) and/or Subgroup (eg, Steering Committee)
Francesco	Dotta			A.O.U. Senese Policlinico "Le Scotte" , U.O.C. Diabetologia	Italy	Principal investigator	
Fiorenzo	Cortinovis			AO Papa Giovanni XXIII USS Dietologia Clinica	Italy	Principal investigator	
Giulia	Cogni			IRCCS Fondazione "S. Maugeri" U.O. Medicina Interna ed Endo	Italy	Principal investigator	
Simona	Bo			A.O.U. Città della Salute e della Scienza di Torino	Italy	Principal investigator	
Lucia	Frittitta			ARNAS Ospedale Garibaldi	Italy	Principal investigator	
Alexis	Malavazos			Centro di Alta Spec. Diet., Educ. Alim. e Prev. Cardio Metab	Italy	Principal investigator	
Marco	Bucci			Università degli studi G. D?Annunzio Chieti Pescara - CAST	Italy	Principal investigator	
Edoardo	Mannucci			AOU Careggi Dipartimento Medico Geriatrico SOD Diabetologia	Italy	Principal investigator	
Paolo	Sbraccia			UOC di Medicina Interna - Centro Medico dell?Obesità	Italy	Principal investigator	
Anna M.	Colao			Azienda Ospedaliera Universitaria Federico II di Napoli	Italy	Principal investigator	
Maria C.	Zatelli			Azienda Osp-Univ Ferrara-Dip Scienze Mediche-Endocrinologia	Italy	Principal investigator	
Fabrizio	Muratori			Ospedale di San Fermo della Battaglia	Italy	Principal investigator	
Frida	Leonetti			Ospedale Santa Maria Goretti - UOD Diabetologia	Italy	Principal investigator	
Dario	Tuccinardi			Pol. Uni. Campus Biomedico UOC Endocrinologia e Diabetologia	Italy	Principal investigator	
Marco	Mirani			Ist.Clinico Humanitas Endocrinologia e Malattie del ricambio	Italy	Principal investigator	

Supplemental Online Content: Nonauthor Collaborators
*First name, last name, and suffix (if applicable) are required and will appear in PubMed.

***First Name and Middle Initial(s)**	***Last Name**	***Suffix (eg, Jr, III)**	Academic Degrees	Institution	Location (city, state/province, country)	Role or Contribution, eg, chair, principal investigator	Group (if more than 1 Group listed in the byline) and/or Subgroup (eg, Steering Committee)
Cesare	Berra			IRCCS Multimedica	Italy	Principal investigator	
Mitsuru	Ohsugi			National Center for Global Health and Medicine	Japan	Principal investigator	
Hideki	Tanaka			Seiwa Clinic	Japan	Principal investigator	
Haruhiko	Onaka			Takatsuki Red Cross Hospital	Japan	Principal investigator	
Atsuyuki	Watanabe			Okayama Medical Center	Japan	Principal investigator	
Toshiyuki	Sugiura			Sugiura Clinic	Japan	Principal investigator	
Kuniaki	Morisaki			Japan Community Health care Organization Ritsurin Hospital	Japan	Principal investigator	
Takuya	Nagano			Kagawa Prefectural Central Hospital	Japan	Principal investigator	
Masayuki	Doi			Kagawa Prefectural Central Hospital, Cardiovascular Medicine	Japan	Principal investigator	
Masahiro	Natsuaki			Saga University Hospital, Department of Cardiology	Japan	Principal investigator	
Naomasa	Miyamoto			Saino Clinic	Japan	Principal investigator	
Kenshi	Fujii			Specified Med.Corp.Watanabeigakukai Sakurabashi Watanabe Hp.	Japan	Principal investigator	
Fumiki	Oh			Shinden Higashi Clinic_Miyagi	Japan	Principal investigator	
Satoru	Mitomo			New Tokyo Heart Clinic_Matsudo-shi, Chiba,	Japan	Principal investigator	
Masahiro	Kawanishi			Ijinkai Takeda General Hospital, Neurosurgery	Japan	Principal investigator	
Ryoji	Kitamura			Ijinkai Takeda General Hospital, Cardiovascular Medicine	Japan	Principal investigator	
Hajime	Maeda			H.E.C Science Clinic	Japan	Principal investigator	
Taiji	Miyake			Gifu Heart Center	Japan	Principal investigator	
Noriyuki	Kinoshita			Koseikai Takeda Hospital	Japan	Principal investigator	
Atsushi	Sato			National Hospital Organization Saitama National Hospital	Japan	Principal investigator	

Supplemental Online Content: Nonauthor Collaborators
*First name, last name, and suffix (if applicable) are required and will appear in PubMed.

*First Name and Middle Initial(s)	*Last Name	*Suffix (eg, Jr, III)	Academic Degrees	Institution	Location (city, state/province, country)	Role or Contribution, eg, chair, principal investigator	Group (if more than 1 Group listed in the byline) and/or Subgroup (eg, Steering Committee)
Yuzo	Takeuchi			Shiga General Hospital	Japan	Principal investigator	
Osamu	Ueda			Chiba Tokushukai Hospital	Japan	Principal investigator	
Arihiro	Kiyosue			Tokyo-Eki Center-building Clinic	Japan	Principal investigator	
Satoshi	Kodera			The Univ. of Tokyo Hp., Dept. of Cardiovascular Medicine	Japan	Principal investigator	
Mamoru	Manita			Naha City Hospital	Japan	Principal investigator	
Yasushi	Fukushima			Fukuwa Clinic	Japan	Principal investigator	
Masaharu	Kinoshita			Nagata Hospital	Japan	Principal investigator	
Masahiro	Yamasaki			Chikamori Hospital	Japan	Principal investigator	
Atsushi	Sueyoshi			Uji Tokushukai Medical Center	Japan	Principal investigator	
Toshiaki	Ando			Kan-etsu chu-oh Hospital	Japan	Principal investigator	
Keiichi	Hanaoka			Hanaoka Seishu Memorial Hospital	Japan	Principal investigator	
Tsuyoshi	Isawa			Sendai Kousei Hospital_Sendai-shi, Miyagi	Japan	Principal investigator	
Yoshiki	Hata			Minamino Cardiovascular Hospital	Japan	Principal investigator	
Hideyuki	Kunishige			Shinsapporo Seiryou Hospital, Intenal Medicine	Japan	Principal investigator	
Mitsutoshi	Oguri			Kasugai Municipal Hospital	Japan	Principal investigator	
Mitsunori	Abe			Medical Corporation Matsuyama-heartcenter Yotsuba Circulatio	Japan	Principal investigator	
Takashi	Matsumoto			Tokyo Shinagawa Hospital Social Medical Corporation Association Tokyokyojuno-kai	Japan	Principal investigator	
Yasuhiro	Ono			Takagi hospital, Internal Medicine	Japan	Principal investigator	
Yoshio	Okada			Toyooka Chuo Hospital	Japan	Principal investigator	
Toshiaki	Kato			Nagoya Ekisaikai Hospital	Japan	Principal investigator	
Tokushi	Koga			Steel Memorial Yawata Hospital	Japan	Principal investigator	
Ryoko	Fujita			Hirakata Kohsai Hospital	Japan	Principal investigator	
Tomohiro	Sakamoto			Saiseikai Kumamoto Hospital	Japan	Principal investigator	

Supplemental Online Content: Nonauthor Collaborators
*First name, last name, and suffix (if applicable) are required and will appear in PubMed.

***First Name and Middle Initial(s)**	***Last Name**	***Suffix (eg, Jr, III)**	Academic Degrees	Institution	Location (city, state/province, country)	Role or Contribution, eg, chair, principal investigator	Group (if more than 1 Group listed in the byline) and/or Subgroup (eg, Steering Committee)
Hideki	Okayama			Ehime Prefectural Central Hospital	Japan	Principal investigator	
Toshiaki	Kadokami			Fukuokaken Saiseikai Futsukaichi Hospital_Cardiovascular Medicine	Japan	Principal investigator	
Hiroshi	Yamaguchi			Hiroshi Yamaguchi Clinic	Japan	Principal investigator	
Takamasa	Iwasawa			Yokosuka General Hospital Uwamachi, Cardiology	Japan	Principal investigator	
Itaru	Maeda			Medical corporation Sanseikai Miyanomori Memorial Hospital	Japan	Principal investigator	
Hiroki	Teragawa			Medical Corporation JR Hiroshima Hospital	Japan	Principal investigator	
Hirotaka	Ezaki			Tokorozawa Heart Center	Japan	Principal investigator	
Arija	Lace			Adoria	Latvia	Principal investigator	
Irina	Veze			Health Centre-4	Latvia	Principal investigator	
Inga	Rezgale			Medical Centre Pulss 5	Latvia	Principal investigator	
Gustavs	Latkovskis			Stradini Cardiology	Latvia	Principal investigator	
Iveta	Sime			Meissana Ltd	Latvia	Principal investigator	
Dace	Teterovska			Teterovska practice	Latvia	Principal investigator	
Gita	Rancane			Ziemelkurzeme Regional Hospital	Latvia	Principal investigator	
Irina	Klavina			Ari Med	Latvia	Principal investigator	
Liew H.	Bang			Hospital Queen Elizabeth II	Malaysia	Principal investigator	
Tiong K.	Ong			Sarawak Heart Centre	Malaysia	Principal investigator	
Saravanan	Krishinan			Hospital Sultanah Bahiyah	Malaysia	Principal investigator	
Li Y.	Lee			Hospital Seri Manjung	Malaysia	Principal investigator	
Norsiah	Ali			Klinik Kesihatan Masjid Tanah	Malaysia	Principal investigator	
Anwar I.	Ruhani			Hospital Tengku Ampuan Afzan	Malaysia	Principal investigator	
Zanariah	Hussein			Hospital Putrajaya	Malaysia	Principal investigator	
Dr A.	Rosman			National Heart Institute	Malaysia	Principal investigator	
Imran B.	Abidin			University Malaya Medical Centre	Malaysia	Principal investigator	
Wan M.	Wan Mohamed			Hospital Universiti Sains Malaysia	Malaysia	Principal investigator	

*First name, last name, and suffix (if applicable) are required and will appear in PubMed.

*First Name and Middle Initial(s)	*Last Name	*Suffix (eg, Jr, III)	Academic Degrees	Institution	Location (city, state/province, country)	Role or Contribution, eg, chair, principal investigator	Group (if more than 1 Group listed in the byline) and/or Subgroup (eg, Steering Committee)
Sazzli S.	Kasim			University Technology MARA (UiTM) - Puncak Alam	Malaysia	Principal investigator	
Enrique C.	Morales Villegas			Centro de Investigación Cardiometabólica de Aguascalientes	Mexico	Principal investigator	
Raymundo	García Reza			Unidad Biomedica Avanzada Monterrey	Mexico	Principal investigator	
José L.	Arenas León			Centro de atención e investigación cardiovascular del Potosí	Mexico	Principal investigator	
José R.	Lazcano Soto			Lahoja Asociación para la Investigación y Farmacovigilancia	Mexico	Principal investigator	
Alberto E.	Bazzoni Ruiz			CIMAB SA de CV	Mexico	Principal investigator	
Pedro A.	Garcia Hernandez			Hospital Universitario Dr. José Eleuterio González_Monterrey	Mexico	Principal investigator	
Silvia A.	Jimenez-Ramos			Centro de Investigacion Clinica Endocrinologica de Jalisco	Mexico	Principal investigator	
Maria A.	Arias Mendoza			Instituto Nacional de Cardiología Ignacio Chavez	Mexico	Principal investigator	
Manuel O.	De Los Rios Ibarra			Centro para el Desarrollo de la Medicina y la Asistencia	Mexico	Principal investigator	
Andre P.	Van Beek			UMC Groningen	Netherlands	Principal investigator	
J.	Schaap			Amphia Ziekenhuis	Netherlands	Principal investigator	
Henk	Swart			D & A Research B.V.	Netherlands	Principal investigator	
F.M.A.C.	Martens			Deventer Ziekenhuis	Netherlands	Principal investigator	
Peter	Nierop			Franciscus Gasthuis en Vlietland	Netherlands	Principal investigator	
T.	Vossenberg			Medisch Centrum Leeuwarden	Netherlands	Principal investigator	
C.	Van Der Zwaan			Ziekenhuis Rivierenland Tiel	Netherlands	Principal investigator	
Reinhart	Dorman			Bravis Ziekenhuis	Netherlands	Principal investigator	
Bjorn	Groenemeijer			Gelre Ziekenhuizen Apeldoorn	Netherlands	Principal investigator	
T.J.	Römer			Alrijne Leiderdorp	Netherlands	Principal investigator	

Supplemental Online Content: Nonauthor Collaborators
*First name, last name, and suffix (if applicable) are required and will appear in PubMed.

*First Name and Middle Initial(s)	*Last Name	*Suffix (eg, Jr, III)	Academic Degrees	Institution	Location (city, state/province, country)	Role or Contribution, eg, chair, principal investigator	Group (if more than 1 Group listed in the byline) and/or Subgroup (eg, Steering Committee)
Joeran	Hjelmesaeth			Senter for sykelig overvekt i Helse Sør-Øst	Norway	Principal investigator	
Serena	Tonstad			Oslo universitetssykehus Aker	Norway	Principal investigator	
Sigrun	Halvorsen			Oslo universitetssykehus HF Ullevål	Norway	Principal investigator	
Lars	Gullestad			Rikshospitalet - Kardiologisk forskning	Norway	Principal investigator	
Tone	Gretland Valderhaug			Akershus Universitetssykehus	Norway	Principal investigator	
Hans O.	Høivik			Falck Norge AS	Norway	Principal investigator	
Rasmus G.	Høgalmen			Sykehuset Innlandet Lillehammer	Norway	Principal investigator	
Ottar K.	Nygård			Haukeland Universitetssykehus	Norway	Principal investigator	
Eva	Rice			Ålesund Sjukehus - Hjertemedisinsk poliklinikk	Norway	Principal investigator	
Pawel	Bogdanski			Centrum Zdrowia Metabolicznego	Poland	Principal investigator	
Edward	Franek			Panstwowy Instytut Medyczny MSWiA	Poland	Principal investigator	
Grzegorz	Gajos			UniCardia & UniMedica & UniEstetica	Poland	Principal investigator	
Lucyna	Ostrowska			Gabinet Leczenia Otylosci i Chorob Dietozaleznych	Poland	Principal investigator	
Ewa	Szyprowska			NZOZ "CenterMed Lublin" Sp. z o.o.	Poland	Principal investigator	
Monika	Lukaszewicz			Centrum Badan Klinicznych PI-House	Poland	Principal investigator	
Grzegorz	Kania			Clinmedica Research sp. z o.o.	Poland	Principal investigator	
Grzegorz	Skoczylas			ETG Warszawa	Poland	Principal investigator	
Monika	Kujawiak			ETG Siedlce	Poland	Principal investigator	
Iwona	Wozniak			KO-MED Centra Kliniczne Sp. z o.o.	Poland	Principal investigator	

*First name, last name, and suffix (if applicable) are required and will appear in PubMed.

***First Name and Middle Initial(s)**	***Last Name**	***Suffix (eg, Jr, III)**	Academic Degrees	Institution	Location (city, state/province, country)	Role or Contribution, eg, chair, principal investigator	Group (if more than 1 Group listed in the byline) and/or Subgroup (eg, Steering Committee)
Monika	Lukaszewicz			Centrum Badan Klinicznych PI-House	Poland	Principal investigator	
Witold	Chrustowski			ETG Lowicz	Poland	Principal investigator	
Malgorzata	Jozefowska			Centrum Terapii Wspolczesnej	Poland	Principal investigator	
Agata	Leksycka			PRATIA Centrum Medyczne	Poland	Principal investigator	
Jaroslaw	Jurowiecki			Poradnia Kardiologiczna Jaroslaw Jurowiecki	Poland	Principal investigator	
Wojciech	Czochra			KO-MED Centra Kliniczne Sp. z o.o., oddzial w Zamosciu	Poland	Principal investigator	
Grazyna	Pulka			GRAZYNA PULKA SPECJALISTYCZNY OSRODEK "ALL-MED"	Poland	Principal investigator	
Agnieszka	Karczmarczyk			Gabinety Lekarskie LabMed	Poland	Principal investigator	
Barbara	Stogowska-Nikiciuk			Trial Medica Dariusz Prochoruk	Poland	Principal investigator	
Piotr	Kalmucki			Centrum Medyczne HCP Sp. z o.o.	Poland	Principal investigator	
Barbara	Stogowska-Nikiciuk			KLIMED LOMZA	Poland	Principal investigator	
Maciej	Beckowski			Przychodnia Lekarzy Specjalistow Serce	Poland	Principal investigator	
Sara	França			Unidade Local de Saúde de Matosinhos	Portugal	Principal investigator	
Luisa	Fonseca			Centro Hospitalar de São João	Portugal	Principal investigator	
Cristina	Martins			Hospital Garcia de Orta	Portugal	Principal investigator	
Pedro	Monteiro			Centro Hospitalar e Universitário de Coimbra	Portugal	Principal investigator	
Fausto	Pinto			Centro Hospitalar Lisboa Norte	Portugal	Principal investigator	
Nicolae	Hancu			S.C. Centrul Medical Unirea S.R.L.	Romania	Principal investigator	
Adrian	Vlad			3rd Clinic for Nutrition-Spitalulul Judetean de Urgenta	Romania	Principal investigator	
Lavinia	Pop			CMI Dr Pop Lavinia	Romania	Principal investigator	

*First name, last name, and suffix (if applicable) are required and will appear in PubMed.

*First Name and Middle Initial(s)	*Last Name	*Suffix (eg, Jr, III)	Academic Degrees	Institution	Location (city, state/province, country)	Role or Contribution, eg, chair, principal investigator	Group (if more than 1 Group listed in the byline) and/or Subgroup (eg, Steering Committee)
Adriana	Onaca			SC Grand Med SRL	Romania	Principal investigator	
Adriana	Cif			SC MEDIAB SRL	Romania	Principal investigator	
Melinda	Kurtinecz			County Emergency Hospital Satu Mare	Romania	Principal investigator	
Ionela M.	Vladu			S.C. TOP DIABET SRL	Romania	Principal investigator	
Ciprian	Constantin			"Carol Davila" Military Emergency Hospital	Romania	Principal investigator	
Valerica	Nafornita			Minimed S.R.L.	Romania	Principal investigator	
Cristina	Toarba			Bella Praxis S.R.L.	Romania	Principal investigator	
Silviana	Constantinescu			S.C. Medcon S.R.L	Romania	Principal investigator	
Rodica	Avram			County Hospital Deva	Romania	Principal investigator	
Georgiana	Nicolescu			SC Gensan SRL, Policlinica ASTRA	Romania	Principal investigator	
Bogdan	Popa			S.C. DIANUTRILIFE MEDICA S.R.L.	Romania	Principal investigator	
Dana	Cosma			S.C. Endodigest S.R.L.	Romania	Principal investigator	
Liliana	Marin			Centrul Medical de Diagnostic si Tratament Ambulatoriu Neomed	Romania	Principal investigator	
Delia V.	Reurean Pintilei			SC Consultmed SRL	Romania	Principal investigator	
Magdalena	Morosanu			SC Diamed Obesity SRL	Romania	Principal investigator	
Nicoleta M.	Mîndrescu			SC NICODIAB SRL	Romania	Principal investigator	
Cristina V.	Mistodie			S.C Milena Sante SRL	Romania	Principal investigator	
Corin V.	Badiu			Institutul National de Endocrinologie C.I. Parhon	Romania	Principal investigator	
Sirona	Lupu			SC Cardiomed SRL	Romania	Principal investigator	
Dan	Enculescu			Sana Monitoring SRL	Romania	Principal investigator	
Elena -.	Caceaune			SC Eco-Diagnosis SRL	Romania	Principal investigator	
Georgiana	Enache			SC Ames Research Center SRL	Romania	Principal investigator	
Ivan G.	Gordeev			Moscow City Clinical Hospital #15	Russian Federation	Principal investigator	
Oksana	Shaydyuk			Medical Center CAPITAL-HEALTH	Russian Federation	Principal investigator	
Zhanna	Kobalava			Friendship University of Russia	Russian Federation	Principal investigator	

Supplemental Online Content: Nonauthor Collaborators
*First name, last name, and suffix (if applicable) are required and will appear in PubMed.

***First Name and Middle Initial(s)**	***Last Name**	***Suffix (eg, Jr, III)**	Academic Degrees	Institution	Location (city, state/province, country)	Role or Contribution, eg, chair, principal investigator	Group (if more than 1 Group listed in the byline) and/or Subgroup (eg, Steering Committee)
Tatiana	Khlevchuk			Moscow City Clinical Hospital n.a. A.K. Eramishantseva	Russian Federation	Principal investigator	
Marat	Ezhov			National Medical Research Center of Cardiology	Russian Federation	Principal investigator	
Viacheslav	Mareev			Moscow State University n.a. M.V. Lomonosov	Russian Federation	Principal investigator	
Olga	Vorobyeva			PHI ?Central Clinical Hospital RZD-Medicine"	Russian Federation	Principal investigator	
Tatiana	Markova			City Clinical Hospital No52	Russian Federation	Principal investigator	
Polina	Ermakova			Astarta Clinic LLC	Russian Federation	Principal investigator	
Olga O.	Bolshakova			FSPbSMU n.a. Academic I.P. Pavlov	Russian Federation	Principal investigator	
Diana	Alpenidze			SPb SBHI City polyclinic #117	Russian Federation	Principal investigator	
Konstantin	Zrazhevskiy			SPb SBHI City Hospital #38 n.a. N.A. Semashko	Russian Federation	Principal investigator	
Ruslan	Sardinov			Saint-Petersburg ?City Policlinic # 74?	Russian Federation	Principal investigator	
Andrey	Obrezan			Consultative & Diagnostic Center with a Outpatient Hospital	Russian Federation	Principal investigator	
Alsu	Zalevskaya			SPb SBHI City Multifield Hospital #2	Russian Federation	Principal investigator	
Olga	Zagrebelnaya			SPb SBHI City Outpatient clinic #37	Russian Federation	Principal investigator	
Irina V.	Dvoryashina			Volosevich First City Clinical Hospital	Russian Federation	Principal investigator	
Dmitriy	Platonov			Regional Clinical Hospital of Tver	Russian Federation	Principal investigator	
Larisa	Khaisheva			Rostov State Medical University_Rostov-on-Don	Russian Federation	Principal investigator	
Elena	Shutemova			Cardiological dispensary	Russian Federation	Principal investigator	
Marina Y.	Sergeeva-Kondrachenko			Penza Regional Clinical Hospital named after N.N. Burdenko	Russian Federation	Principal investigator	
Olga	Smolenskaya			Urals State Medical University	Russian Federation	Principal investigator	

*First name, last name, and suffix (if applicable) are required and will appear in PubMed.

***First Name and Middle Initial(s)**	***Last Name**	***Suffix (eg, Jr, III)**	Academic Degrees	Institution	Location (city, state/province, country)	Role or Contribution, eg, chair, principal investigator	Group (if more than 1 Group listed in the byline) and/or Subgroup (eg, Steering Committee)
Lyudmila A.	Suplotova			Tumen State Medical University	Russian Federation	Principal investigator	
Sergey	Shalaev			Tumen State Medical University - RIMBB	Russian Federation	Principal investigator	
Olga	Gilinskaya			Scientific Research Institute of neuroscience and Medicine	Russian Federation	Principal investigator	
Olga	Barbarash			Institute of Complex Problems of Cardio-Vascular Diseases	Russian Federation	Principal investigator	
Vyacheslav V.	Ryabov			Tomsk National Research Medical Center of the RAS	Russian Federation	Principal investigator	
Alla A.	Boshchenko			Tomsk National Research Medical Center of the RAS	Russian Federation	Principal investigator	
Yulia	Samoilova			Siberian State Medical University_Tomsk	Russian Federation	Principal investigator	
Nadezhda G.	Veselovskaya			RBSHI "Altay Regional Cardiology Dispensary"	Russian Federation	Principal investigator	
Tatyana A.	Lysenko			City Hospital #5	Russian Federation	Principal investigator	
Elena A.	Zhdanova			Vorornezh Regional Clinical Consultive-diagnostic Centre	Russian Federation	Principal investigator	
Mikhail	Zykov			City Hospital #4, Sochi	Russian Federation	Principal investigator	
Lidia	Belousova			Limited Liability Company "Energiya Zdoroviya"	Russian Federation	Principal investigator	
Andrey B.	Peskov			Ulianovsk Regional Clinical Hospital	Russian Federation	Principal investigator	
Irina	Ipatko			SAHI of Komi Republic "Consultative Diagnostic Center"	Russian Federation	Principal investigator	
Irina	Gurieva			Federal Bureau for Medical and Social Expertise	Russian Federation	Principal investigator	
Dmitriy	Ruyatkin			Limited Law Company "Healthy Family" Medicine Center"	Russian Federation	Principal investigator	
Elizaveta	Antonova			Joint Stock Company "Modern Medical Technologies"	Russian Federation	Principal investigator	

Supplemental Online Content: Nonauthor Collaborators
*First name, last name, and suffix (if applicable) are required and will appear in PubMed.

***First Name and Middle Initial(s)**	***Last Name**	***Suffix (eg, Jr, III)**	Academic Degrees	Institution	Location (city, state/province, country)	Role or Contribution, eg, chair, principal investigator	Group (if more than 1 Group listed in the byline) and/or Subgroup (eg, Steering Committee)
Elena E.	Kazakova			Belgorod regional clinical hospital of the St. Ioasaph	Russian Federation	Principal investigator	
Olga	Ershova			Solovyov Clinical Emergency Hospital	Russian Federation	Principal investigator	
Oksana	Vinogradova			Orenburg Regional Clinical Hospital	Russian Federation	Principal investigator	
Galina N.	Guseva			Regional Clinical cardiological dispensary n.a. Polyakov	Russian Federation	Principal investigator	
Yulia	Pergaeva			Reg. State Budget Healthc. Inst. Regional Clinical Hospital	Russian Federation	Principal investigator	
Marina	Kharakhulakh			Tomsk Regional Clinical Hospital	Russian Federation	Principal investigator	
Maxim	Sorokin			LLC RC Medical	Russian Federation	Principal investigator	
Inga	Krivosheeva			LLC Reafan	Russian Federation	Principal investigator	
Albert	Galyavich			KSFMU, Inrereginal Clinical Diagnostic center	Russian Federation	Principal investigator	
Tatiana	Sveklina			SPb SBHI City Outpatient clinic #109	Russian Federation	Principal investigator	
Nebojsa	Lalic			Endocrinology, Diabetes and Metabolism Diseases Clinic	Serbia	Principal investigator	
Katarina	Lalic			Endocrinology, Diabetes and Metabolism Diseases Clinic	Serbia	Principal investigator	
Nemanja	Djenic			Emergency Internal Medicine Clinic	Serbia	Principal investigator	
Marjana	Vukicevic			Special Hospital "Sveti Sava"	Serbia	Principal investigator	
Edita	Stokic			Clin. Centre Vojvodina, Clin. endocr., diab. and met. dis.	Serbia	Principal investigator	
Milika	Asanin			Clinical Centre of Serbia, Emergency Centre, Department of Cardiology	Serbia	Principal investigator	

Supplemental Online Content: Nonauthor Collaborators
*First name, last name, and suffix (if applicable) are required and will appear in PubMed.

***First Name and Middle Initial(s)**	***Last Name**	***Suffix (eg, Jr, III)**	Academic Degrees	Institution	Location (city, state/province, country)	Role or Contribution, eg, chair, principal investigator	Group (if more than 1 Group listed in the byline) and/or Subgroup (eg, Steering Committee)
Goran	Stankovic			Clinical Centre of Serbia, Emergency Centre, Department of Cardiology	Serbia	Principal investigator	
Arsen	Ristic			Clinical centre of Serbia, Clinic for cardiology	Serbia	Principal investigator	
Iftikhar O.	Ebrahim			Dr Iftikhar Osman Ebrahim	South Africa	Principal investigator	
Dorothea	Urbach			Synexus Helderberg Clinical Research Centre	South Africa	Principal investigator	
Clive	Corbett			Dr Corbett	South Africa	Principal investigator	
Landman	Lombard			Cape Town Medical Research Centre	South Africa	Principal investigator	
Aysha	Badat			Wits Bara Clinical Trial Site	South Africa	Principal investigator	
Mohamed	Fulat			Clinical Trial Systems (CTC)	South Africa	Principal investigator	
Cornelia	Kapp			Cardiology Clinical Research	South Africa	Principal investigator	
Vuyokazi N.	Bandezi			CRISMO	South Africa	Principal investigator	
Naeem	Moosa			Dr Moosa's Rooms	South Africa	Principal investigator	
Douwe M.	De Jong			Jongaie Research	South Africa	Principal investigator	
Nqoba	Tsabedze			WITS Clinical Research	South Africa	Principal investigator	
Eugene	Van Der Walt			Roodepoort Medicross Clinical Research Centre	South Africa	Principal investigator	
Elane	Van Nieuwenhuizen			Synexus Watermeyer Clinical Research Centre	South Africa	Principal investigator	
Shahid	Wadvalla			Lenasia Clinical Trial Centre	South Africa	Principal investigator	
Lesley	Burgess			Tread Research (Pty)Ltd	South Africa	Principal investigator	
Kathleen	Coetzee			Paarl Research Centre	South Africa	Principal investigator	
Saleem Y.	Dawood			Dr S Dawood	South Africa	Principal investigator	
Jonathan	Peter			UCT Lung institute	South Africa	Principal investigator	
Louis	Van Zyl			Clinical Projects Research	South Africa	Principal investigator	
Nyda	Fourie			IATROS International	South Africa	Principal investigator	
Johannes J.	Lombaard			Josha Research	South Africa	Principal investigator	

Supplemental Online Content: Nonauthor Collaborators
*First name, last name, and suffix (if applicable) are required and will appear in PubMed.

*First Name and Middle Initial(s)	*Last Name	*Suffix (eg, Jr, III)	Academic Degrees	Institution	Location (city, state/province, country)	Role or Contribution, eg, chair, principal investigator	Group (if more than 1 Group listed in the byline) and/or Subgroup (eg, Steering Committee)
Ellen M.	Makotoko			Cardiology Research_Bloemfontein	South Africa	Principal investigator	
Prakash	Jugnundan			Dr Jugnundan's Rooms	South Africa	Principal investigator	
Zelda E.	Punt			Phoenix Pharma	South Africa	Principal investigator	
Eric	Klug			Tickerdoc Research (Pty) LTD	South Africa	Principal investigator	
Francisco	Tinahones			Hospital Clínico Virgen de la Victoria	Spain	Principal investigator	
Carmen	De Pablo			Hospital Ramón y Cajal	Spain	Principal investigator	
Carmen	De La Cuesta			Clínica Nuevas Tecnologías en Diabetes y Endocrinología	Spain	Principal investigator	
Alfonso	Soto González			Complejo Hospitalario Universitario A Coruña	Spain	Principal investigator	
Juan F.	Merino Torres			Hospital La Fe - Endocrinología y Nutrición	Spain	Principal investigator	
Luis	Masmiquel Comas			Hospital Son Llatzer	Spain	Principal investigator	
Margarita	Rivas Fernández			Hospital Infanta Luisa	Spain	Principal investigator	
Almudena	Castro			Hospital Universitario La Paz	Spain	Principal investigator	
Cristobal	Morales Portillo			Hospital Virgen de la Macarena	Spain	Principal investigator	
Pedro	Mezquita Raya			Centro Periférico de Especialidades Bola Azul	Spain	Principal investigator	
Olga	González Albarrán			Hospital Gregorio Marañón	Spain	Principal investigator	
Lars	Rydén			Karolinska Universitetssjukhuset Solna,FOU Tema Hjärta Kärl	Sweden	Principal investigator	
Björn	Eliasson			Lundberglab for Diab Research	Sweden	Principal investigator	
Helene	Holmer			Centralsjukhuset, Kristianstad	Sweden	Principal investigator	
Thomas	Mooe			Hjärtenheten	Sweden	Principal investigator	

*First name, last name, and suffix (if applicable) are required and will appear in PubMed.

*First Name and Middle Initial(s)	*Last Name	*Suffix (eg, Jr, III)	Academic Degrees	Institution	Location (city, state/province, country)	Role or Contribution, eg, chair, principal investigator	Group (if more than 1 Group listed in the byline) and/or Subgroup (eg, Steering Committee)
Ken	Eliasson			Enheten för Kliniska Studier (EKS), Örebro	Sweden	Principal investigator	
Carl-Johan	Lindholm			Clemenstorgets Hjärtmottagning	Sweden	Principal investigator	
Chau-Chung	Wu			National Taiwan University Hospital	Taiwan, Province of China	Principal investigator	
I-Chang	Hsieh			Chang Gung Memorial Hospital Linko	Taiwan, Province of China	Principal investigator	
Hung-I	Yeh			MacKay Memorial Hospital-Tamsui Branch	Taiwan, Province of China	Principal investigator	
Ming-En	Liu			HsinChu MacKay Memorial Hospital	Taiwan, Province of China	Principal investigator	
Thung-Lip	Lee			E-DA Hosiptal	Taiwan, Province of China	Principal investigator	
Sompongse	Suwanwalaikor n			King Chulalongkorn Memorial Hospital_Bangkok	Thailand	Principal investigator	
Arisara	Suwanagool			Siriraj Hospital_Bangkok_1	Thailand	Principal investigator	
Dilok	Piyayotai			Thammasat University Hospital	Thailand	Principal investigator	
Supawan	Buranapin			Maharaj Nakorn Chiang Mai Hospital	Thailand	Principal investigator	
Prin	Vathesatogkit			Ramathibodi Hospital-Cardiovascular metabolic Center	Thailand	Principal investigator	
Chesda	Udommongkol			Phramongkutklao Hospital-neuro	Thailand	Principal investigator	
Nakarin	Sansanayudh			Phramongkutklao Hospital-cardio	Thailand	Principal investigator	
Songsak	Kiatchoosakun			Srinakarind Hospital-Division of Cardiology	Thailand	Principal investigator	
Wanwarang	Wongcharoen			Maharaj Nakorn Chiang Mai Hospital-cardio	Thailand	Principal investigator	
Selcuk	Dagdelen			Hacettepe Universitesi Tip Fakultesi Hastanesi	Türkiye	Principal investigator	
Yusuf A.	Sonmez			Gulhane Egitim Arastirma Hastanesi	Türkiye	Principal investigator	

Supplemental Online Content: Nonauthor Collaborators
*First name, last name, and suffix (if applicable) are required and will appear in PubMed.

***First Name and Middle Initial(s)**	***Last Name**	***Suffix (eg, Jr, III)**	Academic Degrees	Institution	Location (city, state/province, country)	Role or Contribution, eg, chair, principal investigator	Group (if more than 1 Group listed in the byline) and/or Subgroup (eg, Steering Committee)
Seda	Sancak			Fatih Sultan Mehmet Hastanesi	Türkiye	Principal investigator	
Meral	Kayikcioglu			Ege University School of Medicine, Cardiology Department	Türkiye	Principal investigator	
Mustafa	Unubol			Adnan Menderes Universitesi Uygulama ve Arastirma Hastanesi	Türkiye	Principal investigator	
Ramazan	Sari			Akdeniz University Tip Fakultesi Hastanesi	Türkiye	Principal investigator	
Okan S.	Bakiner			Baskent Universitesi Adana	Türkiye	Principal investigator	
Ahmet	Celik			Mersin University Cardiology	Türkiye	Principal investigator	
Yuksel	Altuntas			Seyrantepe Hamidiye Etfal Egitim ve Arastirma Hastanesi	Türkiye	Principal investigator	
Dilek	Gogas Yavuz			T.C. Saglik Bakanligi Pendik Egitim ve Arastirma Hastanesi	Türkiye	Principal investigator	
Ebru	Ozpelit			Dokuz Eylul University School of Medicine, Cardiology	Türkiye	Principal investigator	
Ibrahim	Gul			Cumhuriyet University School of Medicine-Cardiology	Türkiye	Principal investigator	
Oner	Ozdogan			Izmir Tepecik Training and Research Hospital	Türkiye	Principal investigator	
Dilek	Yazici			Koc University Hospital	Türkiye	Principal investigator	
Mustafa	Araz			Gaziantep Universitesi Tip Fakultesi Hastanesi	Türkiye	Principal investigator	
Mine	Adas			TC SB Ist.il Sag.Müd.Prof.Dr.Cemil Tascioglu Sehir Hastanesi	Türkiye	Principal investigator	
Mazhar M.	Tuna			Umraniye Egitim ve Arastirma Hastanesi	Türkiye	Principal investigator	
Ozlem	Soyluk Selcukbiricik			Istanbul Universitesi Istanbul Tip Fakultesi - Endokrinoloji	Türkiye	Principal investigator	
Mehmet	Kanadasi			Cukurova University School of Medicine Balcali Hospital	Türkiye	Principal investigator	
Abdurrahman	Oguzhan			Erciyes University Cardiology	Türkiye	Principal investigator	

*First name, last name, and suffix (if applicable) are required and will appear in PubMed.

***First Name and Middle Initial(s)**	***Last Name**	***Suffix (eg, Jr, III)**	Academic Degrees	Institution	Location (city, state/province, country)	Role or Contribution, eg, chair, principal investigator	Group (if more than 1 Group listed in the byline) and/or Subgroup (eg, Steering Committee)
Oleksandr	Parkhomenko			MD Strazheska Institute of Cardiology of NAMSU - Resuscitati	Ukraine	Principal investigator	
Zoreslava	Lysak			Oleksandrivska Clinical Hospital - cardio rehabilitation dep	Ukraine	Principal investigator	
Leonid	Rudenko			Kyiv City Clinical Hospital of Emergency Care - Infarction	Ukraine	Principal investigator	
Valentyna	Velychko			Odessa Clinical Railway Hospital - Outpatient department	Ukraine	Principal investigator	
Olena	Levchenko			Odesa Regional Clinical Hospital	Ukraine	Principal investigator	
Yuriy	Mostovoy			City Clinical Hospital #1 - Therapy department	Ukraine	Principal investigator	
Mykola	Stanislavchuk			Vinnytsia Regional Clinical Hospital - Cardiology department	Ukraine	Principal investigator	
Mykola	Kushnir			Zhytomir Regional Clinical Hospital - Cardio-Arrhytm. centre	Ukraine	Principal investigator	
Ivan	Vyshnyvetskyy			Central City Hospital #1 - Scientific-Research center	Ukraine	Principal investigator	
Tetiana	Ilashchuk			CMI City Clinical Hospital #3	Ukraine	Principal investigator	
Olena	Buriakovska			NI of Therapy na LT Malaya of NAMSU - Chr. Non-Communicable	Ukraine	Principal investigator	
Viktoriia	Leonidova			CNPI "Kharkiv City Clinical Hospital #27"	Ukraine	Principal investigator	
Yulian	Kyyak			City Clinical Emergency Hospital - Cardiology department	Ukraine	Principal investigator	
Olha	Horoshko			Kyiv Regional Hospital	Ukraine	Principal investigator	
Zoreslava	Lysak			Medical Center of LLC 'Harmoniya Krasy'	Ukraine	Principal investigator	
Liudmyla	Todoriuk			Medical Center ?Ok!Clinic+? of II of Clinical Studies LLC	Ukraine	Principal investigator	
Galyna	Myshanych			Clinical Hosp on the railway line No2 branch PHC JSC Ukrzaliz	Ukraine	Principal investigator	

Supplemental Online Content: Nonauthor Collaborators
*First name, last name, and suffix (if applicable) are required and will appear in PubMed.

***First Name and Middle Initial(s)**	***Last Name**	***Suffix (eg, Jr, III)**	Academic Degrees	Institution	Location (city, state/province, country)	Role or Contribution, eg, chair, principal investigator	Group (if more than 1 Group listed in the byline) and/or Subgroup (eg, Steering Committee)
John	Wilding			University Hospital Aintree	United Kingdom	Principal investigator	
Robert	Andrews			Musgrove Park Hospital	United Kingdom	Principal investigator	
N?jaimeh	Asamoah			Crouch Oak Family Practice	United Kingdom	Principal investigator	
Mark	Barber			Stroke Trials Office	United Kingdom	Principal investigator	
Srikanth	Bellary			Birmingham Heartlands Hospital	United Kingdom	Principal investigator	
Andrew	Broadley			Clinical Research Unit	United Kingdom	Principal investigator	
Matt	Capehorn			Clifton Medical Centre	United Kingdom	Principal investigator	
Rebecca	Clark			Layton Medical Centre	United Kingdom	Principal investigator	
Piers	Clifford			Wycombe General Hospital	United Kingdom	Principal investigator	
Jeff	Stoker			Bermuda Practice	United Kingdom	Principal investigator	
Steven	Creely			Royal Cornwall Hospital (Treliske)	United Kingdom	Principal investigator	
Cuong	Dang			Diabetes Centre North Manchester General Hospital	United Kingdom	Principal investigator	
Simon	Dobson			Clarence Medical Centre	United Kingdom	Principal investigator	
Patrick	English			University Dept of Medicine, Derriford Hospital, Plymouth	United Kingdom	Principal investigator	
Ewart	Jackson-Voyzey			Axbridge & Wedmore Medical Practice	United Kingdom	Principal investigator	
Danijela	Tatovic			Southmead Hospital	United Kingdom	Principal investigator	
Philip	Keeling			Cardiology Department	United Kingdom	Principal investigator	
Jeffrey	Khoo			Cardiology	United Kingdom	Principal investigator	
Mary	Lynch			Lister Hospital	United Kingdom	Principal investigator	
Kelvin	Lee			Lincoln County Hospital	United Kingdom	Principal investigator	
Barbara	Mcgowan			Guys Hospital	United Kingdom	Principal investigator	
Yaswin	Beerachee			Harrogate District Hospital	United Kingdom	Principal investigator	
Alexander	Miras			Imperial College London	United Kingdom	Principal investigator	
Min	Myint			Sunderland Royal Hospital	United Kingdom	Principal investigator	
Sunil	Nair			Countess Of Chester NHS Foundation Trust	United Kingdom	Principal investigator	
Neil	Paul			Ashfields Primary Care Centre	United Kingdom	Principal investigator	
Hermione	Price			Moorgreen Hospital	United Kingdom	Principal investigator	

Supplemental Online Content: Nonauthor Collaborators
*First name, last name, and suffix (if applicable) are required and will appear in PubMed.

*First Name and Middle Initial(s)	*Last Name	*Suffix (eg, Jr, III)	Academic Degrees	Institution	Location (city, state/province, country)	Role or Contribution, eg, chair, principal investigator	Group (if more than 1 Group listed in the byline) and/or Subgroup (eg, Steering Committee)
Harpal	Randeva			WISDEM Centre	United Kingdom	Principal investigator	
Thozhukat	Sathyapalan			Hull Royal Infirmary	United Kingdom	Principal investigator	
Roxy	Senior			Department Of Cardiac Research	United Kingdom	Principal investigator	
Raj	Sharma			Sea Road Surgery	United Kingdom	Principal investigator	
Sumeet	Sharma			St Peters Hospital, Chertsey	United Kingdom	Principal investigator	
Anna	Strzelecka			Antrim Area Hospital	United Kingdom	Principal investigator	
Neil	Swanson			Cardio Research	United Kingdom	Principal investigator	
Graeme	Tait			University Hospital Wishaw	United Kingdom	Principal investigator	
Bijay	Vaidya			Royal Devon University Healthcare NHS Foundation Trust	United Kingdom	Principal investigator	
Royce	Vincent			Kings College Hospital	United Kingdom	Principal investigator	
John	Wakeling			Ely Bridge Surgery	United Kingdom	Principal investigator	
Pete	Wilson			Bollington Medical Centre	United Kingdom	Principal investigator	
Yuk-Ki	Wong			St. Richards Hospital	United Kingdom	Principal investigator	
Nell	Wyatt			The Health Centre	United Kingdom	Principal investigator	
John M.	Agaiby			Clinical Inv Spec, Inc.Kenosha	United States	Principal investigator	
Amer	Al-Karadsheh			The Endocrine Center_Houston	United States	Principal investigator	
Caroline	Apovian			Boston Medical Center_Cary	United States	Principal investigator	
Ahmed A.	Arif			AA MRC LLC	United States	Principal investigator	
Vanita R.	Aroda			Brigham & Women's Hospital	United States	Principal investigator	
Heidi C.	Shea			Research Institute Of Dallas	United States	Principal investigator	
Otis	Barnum			Barnum Medical Research, Inc.	United States	Principal investigator	
Martin	Berk			Texas Health Physicians Group_Dallas	United States	Principal investigator	
Isabel	Pereira			Synexus Clinical Research US, Inc._Horsham	United States	Principal investigator	
William C.	Biggs			Amarillo Med Spec LLP	United States	Principal investigator	
Liana	Billings			NorthShore Univ Hlth Sys	United States	Principal investigator	
Donald	Brautigam			Great Lakes Medical Research	United States	Principal investigator	
Andrew P.	Brockmyre			Holston Medical Group Pc	United States	Principal investigator	
Matthew J.	Budoff			Lundquist Inst-Biomed Innovtn	United States	Principal investigator	

*First name, last name, and suffix (if applicable) are required and will appear in PubMed.

***First Name and Middle Initial(s)**	***Last Name**	***Suffix (eg, Jr, III)**	Academic Degrees	Institution	Location (city, state/province, country)	Role or Contribution, eg, chair, principal investigator	Group (if more than 1 Group listed in the byline) and/or Subgroup (eg, Steering Committee)
Barry	Buffman			Quartz Hill Walk-in Medical Group, inc.	United States	Principal investigator	
Bartolomé	Burguera			Cleveland Clinic_Cleveland	United States	Principal investigator	
Robert	Busch			Albany Medical College ? Endo	United States	Principal investigator	
John B.	Buse			UNC Eastowne Clinical Trials Unit	United States	Principal investigator	
Robert S.	Call			Clinical Research Partners, LLC	United States	Principal investigator	
Kevin	Cannon			Accellacare	United States	Principal investigator	
Brian	Curtis			Corvallis Clinic PC Clinical Research Department	United States	Principal investigator	
Ronald H.	Chochinov			Coastal Metabolic Research Center	United States	Principal investigator	
Cornell V.	Calinescu			Synexus Clinical Research US	United States	Principal investigator	
Wayne	Clark			Oregon Health & Science Uni	United States	Principal investigator	
Matthew	Lewis			New West Physicians,Inc.	United States	Principal investigator	
Bradley P.	Swenson			Synexus Clinical Research US, Inc._Anderson	United States	Principal investigator	
George M.	Cornett			American Health Network of IN LLC_Franklin	United States	Principal investigator	
Nizar Y.	Daboul			Advanced Med Res Maumee	United States	Principal investigator	
Cyrus V.	Desouza			University of NE Med Ctr	United States	Principal investigator	
Nediljka	Buljubasic			VAGLAHS Clinical Research Center (CRC)	United States	Principal investigator	
Matthew P.	Finneran			New Venture Medical Research	United States	Principal investigator	
Steven	Fordan			Thyroid, Endocrinology, and Diabetes, PA	United States	Principal investigator	
Jr. W.	Gandy			Ellipsis Group	United States	Principal investigator	
William T.	Garvey			Univ of Alabama Birmingham	United States	Principal investigator	
Steven A.	Geller			Centennial Medical Group	United States	Principal investigator	
Jeffrey G.	Geohas			Evanston Premier Hlthcr Res	United States	Principal investigator	
Matthew P.	Gilbert			U. of Vermont Med Ctr Burlington	United States	Principal investigator	
Carl E.	Dukes			Sun Research Institute	United States	Principal investigator	

Supplemental Online Content: Nonauthor Collaborators
*First name, last name, and suffix (if applicable) are required and will appear in PubMed.

***First Name and Middle Initial(s)**	***Last Name**	***Suffix (eg, Jr, III)**	Academic Degrees	Institution	Location (city, state/province, country)	Role or Contribution, eg, chair, principal investigator	Group (if more than 1 Group listed in the byline) and/or Subgroup (eg, Steering Committee)
Aaron N.	Hartman			Virginia Research Center	United States	Principal investigator	
Lee	Herman			Herman Clinical Research LLC	United States	Principal investigator	
Patricia M.	Houser			Amherst Family Practice P.C.	United States	Principal investigator	
Moises	Issa			Clinical Research of Hollywood_Hollywood	United States	Principal investigator	
Richard A.	Jackson			Dominion Medical Associates	United States	Principal investigator	
Michael F.	Jardula			Desert Oasis Hlthcr Med Group	United States	Principal investigator	
Terri	Jerkins			MidState Endocrine Associates	United States	Principal investigator	
John M.	Joyce			Clinical Neuroscience Solutions, Inc.	United States	Principal investigator	
Richard	Kastelic			Richard M Kastelic MD Assoc	United States	Principal investigator	
Bindu M.	Nayak			Wenatchee Valley Hospital and Clinics	United States	Principal investigator	
Dean J.	Kereiakes			The Carl & Edyth Lindner Center	United States	Principal investigator	
Eric J.	Klein			Capital Clin Res Ctr,LLC	United States	Principal investigator	
Ted	Kubicki			Sentara Medical Group_Norfolk	United States	Principal investigator	
Gilbert N.	Ledesma			Arlington Family Research Center, Inc.	United States	Principal investigator	
Omari	Ruffin			Trinity Clinical Research LLC	United States	Principal investigator	
Thomas	Lenzmeier			Synexus Clinical Research	United States	Principal investigator	
Norman	Lepor			National Heart Institute Cal	United States	Principal investigator	
Jenny	Liu			UniMed Center	United States	Principal investigator	
Holly	Lofton			NYU Grossman School of Med	United States	Principal investigator	
Charles F.	Lovell			York Clinical Research LLC	United States	Principal investigator	
Steven P.	Marso			Midwest Heart & Vasc Spec	United States	Principal investigator	
Eric M.	Folkens			Bradenton Research Center	United States	Principal investigator	
Barry K.	Mclean			Central Alabama Research	United States	Principal investigator	
Peter	Meyers			ClinSite LLC.	United States	Principal investigator	
Avinash	Murthy			Central Illinois Diabetes and Clinical Research	United States	Principal investigator	

Supplemental Online Content: Nonauthor Collaborators
*First name, last name, and suffix (if applicable) are required and will appear in PubMed.

***First Name and Middle Initial(s)**	***Last Name**	***Suffix (eg, Jr, III)**	Academic Degrees	Institution	Location (city, state/province, country)	Role or Contribution, eg, chair, principal investigator	Group (if more than 1 Group listed in the byline) and/or Subgroup (eg, Steering Committee)
Wallace	Johnson			University Of Maryland School of Medicine	United States	Principal investigator	
Samer N.	Nakhle			Palm Research Center, Inc.	United States	Principal investigator	
Philip	O'donnell			Selma Medical Associates	United States	Principal investigator	
Alexander	Perkelvald			Dr. Alexander Perkelvald	United States	Principal investigator	
Athena	Philis-Tsimikas			Scripps Whittier Diabetes Inst	United States	Principal investigator	
Talessa	Powell			American Health Network of Indiana, LLC_Greenfield	United States	Principal investigator	
Richard E.	Pratley			AdventHealth Diab Inst	United States	Principal investigator	
Raman S.	Purighalla			Preferred Primary Care Physicians Inc.	United States	Principal investigator	
Marina A.	Raikhel			Torrance Clin Res Inst, Inc.	United States	Principal investigator	
Neda	Rasouli			University of Colorado Hospital	United States	Principal investigator	
Jeffrey	Zacher			Synexus Cln Rsrch /Cnt Phoenix Med C	United States	Principal investigator	
Joseph A.	Risser			San Diego Family Care	United States	Principal investigator	
Marcelo	Correia			University of Iowa Prev Inter Ctr	United States	Principal investigator	
Glenn	Rosen			Parkside Family Medicine	United States	Principal investigator	
Aditya K.	Samal			Northwest Houston Cardiology, P.A.	United States	Principal investigator	
William	Sargeant			Healthscan Clinical Trials,LLC.	United States	Principal investigator	
Airani	Sathananthan			Western University of Health Sciences	United States	Principal investigator	
Robert J.	Anderson			VA NEB - Western IA Health Stm	United States	Principal investigator	
Yshay	Shlesinger			NorCal Endocrinology and Internal Medicine	United States	Principal investigator	
John	Poremba			Southern NH Diabetes and Endocrinology_Nashua	United States	Principal investigator	
Elias	Siraj			Strelitz Diabetes Of Eastern V.	United States	Principal investigator	
Kanagaratnam	Sivalingam			First Valley Medical Group	United States	Principal investigator	
Stephen K.	Smith			Burke Primary Care	United States	Principal investigator	

*First name, last name, and suffix (if applicable) are required and will appear in PubMed.

***First Name and Middle Initial(s)**	***Last Name**	***Suffix (eg, Jr, III)**	Academic Degrees	Institution	Location (city, state/province, country)	Role or Contribution, eg, chair, principal investigator	Group (if more than 1 Group listed in the byline) and/or Subgroup (eg, Steering Committee)
Brian	Snyder			Southgate Medical Group, LLP	United States	Principal investigator	
Ryan	Murray			WakeMed Garner Hlthplx	United States	Principal investigator	
C. W.	Sofley			Internal Medicine Associates of Anderson	United States	Principal investigator	
Christopher	Still			Geisinger Clinic	United States	Principal investigator	
Joshua B.	Oaks			Wade Family Medicine	United States	Principal investigator	
Mark L.	Warren			Physician's East Endocrinology	United States	Principal investigator	
Kathleen	Harris			DCOL Ctr for Clin Res	United States	Principal investigator	
Craig	Wierum			InvestiClin Research	United States	Principal investigator	
Alan	Wynne			Cotton O'Neil Clin Research Ctr	United States	Principal investigator	
Suraj	Saggar			Holy Name Medical Center	United States	Principal investigator	
Tira	Chaicha-Brom			Texas Diab & Endo, P.A.	United States	Principal investigator	
Qingyang	Yuan			Hospital of the Univ PA	United States	Principal investigator	
David	Klonoff			Mills-Peninsula Medical Center	United States	Principal investigator	
Minesh B.	Patel			Franciscan Health Michigan City	United States	Principal investigator	
Narendra	Singh			NSC Research, Inc	United States	Principal investigator	
Senan	Sultan			Northeast Research Institute	United States	Principal investigator	
Juan C.	Garza			VIP Trials	United States	Principal investigator	
Robert P.	Morin			Quality Research Inc	United States	Principal investigator	
Thaddeus H.	Riley			Family Health Care Center	United States	Principal investigator	
Ayham	Shneker			San Antonio Prem Int Med	United States	Principal investigator	
Ania	Jastreboff			Yale University School Of Medicine	United States	Principal investigator	
James	Wallace			Palmetto Clinical Research	United States	Principal investigator	
Jonathan	Condit			American Health Network of IN LLC	United States	Principal investigator	
Michael J.	Koren			Jacksonville Ctr For Clin Res	United States	Principal investigator	
Iqbal	Munir			Riverside University Health System Medical Center	United States	Principal investigator	
Bruce	Weber			Macoupin Research Group	United States	Principal investigator	
Naseem A.	Jaffrani			Cambridge Medical Trials	United States	Principal investigator	

Supplemental Online Content: Nonauthor Collaborators
*First name, last name, and suffix (if applicable) are required and will appear in PubMed.

***First Name and Middle Initial(s)**	***Last Name**	***Suffix (eg, Jr, III)**	Academic Degrees	Institution	Location (city, state/province, country)	Role or Contribution, eg, chair, principal investigator	Group (if more than 1 Group listed in the byline) and/or Subgroup (eg, Steering Committee)
Eric	Hewitt			American Health Network of Indiana, LLC_Avon_0	United States	Principal investigator	
Maja	Delibasic			Mercy Hospital and Medical Center	United States	Principal investigator	
Steven	Lupovitch			Northwest Heart Clinical Research, LLC	United States	Principal investigator	
Ashwini K.	Davuluri			Baptist Heart Specialists_Jacksonville	United States	Principal investigator	
Sathya S.	Krishnasamy			Robley Rex VA Medical Center	United States	Principal investigator	
Kathleen	Ward			Lemah Creek Clinical Research	United States	Principal investigator	
Carlos	Sotolongo			Baptist Heart Specialists_Jacksonville Beach	United States	Principal investigator	
Mauricio	Concha			Intercoastal Medical Group	United States	Principal investigator	
Azazuddin	Ahmed			Apex Medical Research Inc	United States	Principal investigator	
Jeffrey	Carr			Christus Heart and Vascular Institute	United States	Principal investigator	
Brian	Heimer			American Health Network of Indiana, LLC	United States	Principal investigator	
Stuart	Zarich			Bridgeport Hospital	United States	Principal investigator	
Emily J.	Morawski			Holston Medical Group	United States	Principal investigator	
Ranganatha P.	Potu			Nature Coast Clin Rsrch_Crystal River	United States	Principal investigator	
Richard	Lorraine			Harleysville Medical Associates	United States	Principal investigator	
Maher	Agha			OnSite Clinical Solutions, LLC	United States	Principal investigator	
Mehrdad	Ariani			Valley Clinical Trials, Inc.	United States	Principal investigator	
Wa'el	Bakdash			Community Clinical Research_Anderson	United States	Principal investigator	
Marc	Bernstein			Louisiana Heart Center	United States	Principal investigator	
Cristian F.	Breton			International Research Associates, LLC_Miami	United States	Principal investigator	

*First name, last name, and suffix (if applicable) are required and will appear in PubMed.

*First Name and Middle Initial(s)	*Last Name	*Suffix (eg, Jr, III)	Academic Degrees	Institution	Location (city, state/province, country)	Role or Contribution, eg, chair, principal investigator	Group (if more than 1 Group listed in the byline) and/or Subgroup (eg, Steering Committee)
Alexander	Dela Llana			MediSphere Medical Research Center, LLC	United States	Principal investigator	
Paul J.	Opsahl			Providence Health Partners Ctr	United States	Principal investigator	
Barry	Harris			Integrative Research Associates, Inc	United States	Principal investigator	
Bruce	Iteld			Louisiana Heart Center_Slidell	United States	Principal investigator	
Hiba	Abou Assi			Duke University_Durham	United States	Principal investigator	
Timothy	Logemann			Aspirus Research Institute	United States	Principal investigator	
Imran A.	Siddiqui			Simcare Medical Research, LLC	United States	Principal investigator	
Stephen	Ong			MD Medical Research	United States	Principal investigator	
Elba A.	Perez-Vargas			San Miguel Medical	United States	Principal investigator	
Renee	Sangrigoli			Doylestown Hospital Pharmacy	United States	Principal investigator	
Howard S.	Wenocur			Tristar Clin Investigations, PC	United States	Principal investigator	
Louis J.	Levy			TriWest Research Associates	United States	Principal investigator	
David D.	Mcpherson			UT Health University of Texas	United States	Principal investigator	
Bradley	Block			Oviedo Medical Research, LLC	United States	Principal investigator	
Sidney C.	Gorton			Belzoni Clinical Research	United States	Principal investigator	
Mansoor	Qureshi			Trinity Health Michigan Heart	United States	Principal investigator	
Felix	Sigal			Angel City Research, Inc.	United States	Principal investigator	
Devjit	Tripathy			AUDIE L. MURPHY VA HOSPITAL, STVHCS	United States	Principal investigator	
Steven K.	Brodie			Optumcare Colorado Springs, LLC	United States	Principal investigator	
Anthony	Vo			Long Beach VA healthcare System	United States	Principal investigator	
Howard	Weintraub			NYU School of Medicine	United States	Principal investigator	
Jonathan P.	Wilson			PMG Research of Winston-Salem	United States	Principal investigator	
Louis J.	Aronne			Comprehensive Weight Ctrl Prog	United States	Principal investigator	
Hanid	Audish			Encompass Clinical Research_Spring Valley	United States	Principal investigator	
Seth	Baum			Excel Med Ctr Clinical Trials	United States	Principal investigator	
Isaac	Beshay			FDRC	United States	Principal investigator	
William	Herzog			Duke University - S.E Cardio	United States	Principal investigator	

Supplemental Online Content: Nonauthor Collaborators
*First name, last name, and suffix (if applicable) are required and will appear in PubMed.

***First Name and Middle Initial(s)**	***Last Name**	***Suffix (eg, Jr, III)**	Academic Degrees	Institution	Location (city, state/province, country)	Role or Contribution, eg, chair, principal investigator	Group (if more than 1 Group listed in the byline) and/or Subgroup (eg, Steering Committee)
Danish	Jabbar			Amicis Centers of Clinical Research	United States	Principal investigator	
Peter	Levins			Synexus Clinical Research US, Inc./Tatum	United States	Principal investigator	
Gary	Miller			Cardiology Consultants of Danville Inc.	United States	Principal investigator	
Richard E.	Mills			Accellacare US Inc._SC	United States	Principal investigator	
Patrick	Oneil			Medical Uni of SC Charleston	United States	Principal investigator	
Rodica	Pop-Busui			Michigan Medicine	United States	Principal investigator	
John	Pullman			Mercury Street Medical Group, PLLC_Butte	United States	Principal investigator	
David	Robbins			The University Of Kansas Medical Center - Cray Dia	United States	Principal investigator	
David G.	Robertson			Atlanta Diabetes Associates	United States	Principal investigator	
Helena W.	Rodbard			Endo And Metab Cons	United States	Principal investigator	
Royce	Solano			Optumcare Colorado Springs, LLC_Monument	United States	Principal investigator	
Joseph	Soufer			Chase Medical Research LLC	United States	Principal investigator	
Aparna	Tamirisa			Houston Center for Clinical Research LLC	United States	Principal investigator	
Lisa	Usdan			Clinical Neuroscience Solutions	United States	Principal investigator	
Joseph	Lash			Norton Clinical Research Group	United States	Principal investigator	
Jesse A.	Doran			Maryland Cardiovascular Spec	United States	Principal investigator	
Hayden	Bosworth			Durham VA Medical Center - Duke University	United States	Principal investigator	
Ali	Iranmanesh			VA Medical Center_Salem	United States	Principal investigator	
Jennifer	Sidey			Fleming Island Center for Clinical Research	United States	Principal investigator	
Frank	Mcgrew			The Stern Cardiovascular Group	United States	Principal investigator	
Ciro R.	Reyes			Reyes Clinical Research, Inc	United States	Principal investigator	
Michael L.	Gambill			Bayview Physician Services	United States	Principal investigator	

Supplemental Online Content: Nonauthor Collaborators
*First name, last name, and suffix (if applicable) are required and will appear in PubMed.

***First Name and Middle Initial(s)**	***Last Name**	***Suffix (eg, Jr, III)**	Academic Degrees	Institution	Location (city, state/province, country)	Role or Contribution, eg, chair, principal investigator	Group (if more than 1 Group listed in the byline) and/or Subgroup (eg, Steering Committee)
Ronald	D'agostino			Long Island Cardiovascular Consultants PC	United States	Principal investigator	
James	Welker			Anne Arundel Medical Center_Annapolis	United States	Principal investigator	
Henry	Tran			Inova Cardiology-Fairfax	United States	Principal investigator	
David	Kandath			Saratoga Clinical Research	United States	Principal investigator	
Harold E.	Bays			L-MARC Research Center	United States	Principal investigator	
Robert	Buynak			Buynak Clinical Research, P.C.	United States	Principal investigator	
Rodolfo	Carrillo-Jimenez			Florida Premier Cardiology	United States	Principal investigator	
Cuper	Martinez			Kalo Clinical Research	United States	Principal investigator	
Fahed	Bitar			Valley Clinical Trials	United States	Principal investigator	
Adline	Ghazi			MedStar Good Samaritan Hosp	United States	Principal investigator	
Arti	Bhan			Henry Ford Health System	United States	Principal investigator	
John	Scott			National Clin Res Inc.	United States	Principal investigator	
Harvey	Serota			St Louis Heart & Vascular, P.C.	United States	Principal investigator	
Edward S.	Pereira			Est Cst Inst for Rsrch,Jksnvil	United States	Principal investigator	
Keith	Friedman			Johns Hopkins University	United States	Principal investigator	
Dale C.	Allison			Hillcrest Family Health Center	United States	Principal investigator	
Sandeep	Chandra			Atlanta Heart Specialists	United States	Principal investigator	
Aliaksandr	Trusau			St. Vincent Hosp -Prevea Health	United States	Principal investigator	
Neerav	Shah			Cardio Partners Clin Res Inst	United States	Principal investigator	
Suhail	Khadra			Chicago Medical Research LLC	United States	Principal investigator	
Douglas	Spriggs			Clearwater Cardiovascular Consultants	United States	Principal investigator	
Harvey	Serota			St Louis Heart & Vascular, P.C.	United States	Principal investigator	
