# Supplementary material for: Semaglutide on liver fibrosis and heart outcomes in patients at high risk of liver fibrosis: a prespecified analysis of the SELECT randomized trial
Source: Nat Med. 2026 Apr 2;32(5):1686–93. doi: 10.1038/s41591-026-04281-1 (PMC13190271; doi:10.1038/s41591-026-04281-1)
Supplement: Supplementary file 2 — Reporting Summary [file 41591_2026_4281_MOESM2_ESM.pdf]

nature portfolio

Corresponding author(s): Sebastian Meyhöfer

Last updated by author(s): Jan 27, 2026

Reporting Summary

Nature Portfolio wishes to improve the reproducibility of the work that we publish. This form provides structure for consistency and transparency in reporting. For further information on Nature Portfolio policies, see our Editorial Policies and the Editorial Policy Checklist.

Statistics

For all statistical analyses, confirm that the following items are present in the figure legend, table legend, main text, or Methods section.

n/a	Confirmed	
☐	☒	The exact sample size (*n*) for each experimental group/condition, given as a discrete number and unit of measurement
☒	☐	A statement on whether measurements were taken from distinct samples or whether the same sample was measured repeatedly
☐	☒	The statistical test(s) used AND whether they are one- or two-sided<br>*Only common tests should be described solely by name; describe more complex techniques in the Methods section.*
☐	☒	A description of all covariates tested
☐	☒	A description of any assumptions or corrections, such as tests of normality and adjustment for multiple comparisons
☐	☒	A full description of the statistical parameters including central tendency (e.g. means) or other basic estimates (e.g. regression coefficient) AND variation (e.g. standard deviation) or associated estimates of uncertainty (e.g. confidence intervals)
☐	☒	For null hypothesis testing, the test statistic (e.g. *F*, *t*, *r*) with confidence intervals, effect sizes, degrees of freedom and *P* value noted<br>*Give P values as exact values whenever suitable.*
☒	☐	For Bayesian analysis, information on the choice of priors and Markov chain Monte Carlo settings
☐	☒	For hierarchical and complex designs, identification of the appropriate level for tests and full reporting of outcomes
☒	☐	Estimates of effect sizes (e.g. Cohen's *d*, Pearson's *r*), indicating how they were calculated

Our web collection on statistics for biologists contains articles on many of the points above.

Software and code

Policy information about availability of computer code

Data collection	*Provide a description of all commercial, open source and custom code used to collect the data in this study, specifying the version used OR state that no software was used.*
Data analysis	All statistical analyses were performed with SAS software, version 9.4 (SAS Institute)

For manuscripts utilizing custom algorithms or software that are central to the research but not yet described in published literature, software must be made available to editors and reviewers. We strongly encourage code deposition in a community repository (e.g. GitHub). See the Nature Portfolio guidelines for submitting code & software for further information.

Data

Policy information about availability of data

All manuscripts must include a data availability statement. This statement should provide the following information, where applicable:

- Accession codes, unique identifiers, or web links for publicly available datasets
- A description of any restrictions on data availability
- For clinical datasets or third party data, please ensure that the statement adheres to our policy

Bona fide researchers may request access to data by submitting a research proposal for approval by Novo Nordisk and an independent review panel. Data sharing requests may be considered after research completion and primary publication, or, if the trial is intended for regulatory submission, after approval of product and

product use in both the EU and USA. Individual participant data will be shared in data sets in anonymised format. Information about the data sharing process can be found at novonordisk-trials.com.

Research involving human participants, their data, or biological material

Policy information about studies with human participants or human data. See also policy information about sex, gender (identity/presentation), and sexual orientation and race, ethnicity and racism.

Reporting on sex and gender	Biological sex was reported for trial participants; information on gender was not collected.
Reporting on race, ethnicity, or other socially relevant groupings	Race and ethnicity were self-reported for trial participants and can be found in the primary publication (Lincoff AM et al. NEJM 389; 2221-2232 (2023).
Population characteristics	Population characteristics are reported in Table 1; additional characteristics can be found in the primary publication (Lincoff AM et al. NEJM 389; 2221-2232 (2023).
Recruitment	Detailed in the primary publication (Lincoff AM et al. NEJM 389; 2221-2232 (2023).
Ethics oversight	National and institutional regulatory and ethical authorities approved the protocol, and all patients provided written and informed consent. There is no separate ethical approval for this pre-specified secondary analysis of the SELECT trial, as ethical approval has been obtained already (Lincoff AM et al. N Engl J Med 2023; 389:2221-32).

Note that full information on the approval of the study protocol must also be provided in the manuscript.

Field-specific reporting

Please select the one below that is the best fit for your research. If you are not sure, read the appropriate sections before making your selection.

☒ Life sciences ☐ Behavioural & social sciences ☐ Ecological, evolutionary & environmental sciences

For a reference copy of the document with all sections, see nature.com/documents/nr-reporting-summary-flat.pdf

Life sciences study design

All studies must disclose on these points even when the disclosure is negative.

Sample size	17,604. Sample size determination was provided in the primary publication (Lincoff AM et al. NEJM 389; 2221-2232 (2023).
Data exclusions	No data were excluded.
Replication	This is an international, randomized, double-blind trial; thus replication is not possible.
Randomization	Participants were randomized in a 1:1 ratio.
Blinding	Participants and investigators were blinded to treatment allocation.

Reporting for specific materials, systems and methods

We require information from authors about some types of materials, experimental systems and methods used in many studies. Here, indicate whether each material, system or method listed is relevant to your study. If you are not sure if a list item applies to your research, read the appropriate section before selecting a response.

Materials & experimental systems

n/a	Involved in the study	
☒	☐	Antibodies
☒	☐	Eukaryotic cell lines
☒	☐	Palaeontology and archaeology
☒	☐	Animals and other organisms
☐	☒	Clinical data
☒	☐	Dual use research of concern
☒	☐	Plants

Methods

n/a	Involved in the study	
☒	☐	ChIP-seq
☒	☐	Flow cytometry
☒	☐	MRI-based neuroimaging

Clinical data

Policy information about clinical studies

All manuscripts should comply with the ICMJE guidelines for publication of clinical research and a completed CONSORT checklist must be included with all submissions.

Clinical trial registration	NCT03574597
Study protocol	Provided in the primary publication (Lincoff AM et al. NEJM 389; 2221-2232 (2023).
Data collection	The SELECT study enrolled participants from 41 countries between October 2018 through March 2021. All potentially eligible patients were eligible to take part, thereby minimizing any potential self-selection bias.
Outcomes	All of the primary and secondary outcomes, and the statistical methodologies used to analyze them, were prespecified in the Statistical Analysis Plan. All statistical analyses performed for all outcome measures were thus predefined.

Plants

Seed stocks	*Report on the source of all seed stocks or other plant material used. If applicable, state the seed stock centre and catalogue number. If plant specimens were collected from the field, describe the collection location, date and sampling procedures.*
Novel plant genotypes	*Describe the methods by which all novel plant genotypes were produced. This includes those generated by transgenic approaches, gene editing, chemical/radiation-based mutagenesis and hybridization. For transgenic lines, describe the transformation method, the number of independent lines analyzed and the generation upon which experiments were performed. For gene-edited lines, describe the editor used, the endogenous sequence targeted for editing, the targeting guide RNA sequence (if applicable) and how the editor was applied.*
Authentication	*Describe any authentication procedures for each seed stock used or novel genotype generated. Describe any experiments used to assess the effect of a mutation and, where applicable, how potential secondary effects (e.g. second site T-DNA insertions, mosiacism, off-target gene editing) were examined.*
